# Non-Invasive Brain Stimulation for Amyotrophic Lateral Sclerosis: Current Evidence and Future Perspectives

**DOI:** 10.3390/medicina61091685

**Published:** 2025-09-17

**Authors:** Jacopo Della Toffola, Edoardo Ricci, Magda Quagliotto, Paolo Manganotti, Alberto Benussi

**Affiliations:** Neurology Unit, Department of Medical, Surgical and Health Sciences, University of Trieste, 34149 Trieste, Italy; jacopo.dellatoffola@studenti.units.it (J.D.T.); edoardo.ricci@studenti.units.it (E.R.); magda.quagliotto@studenti.units.it (M.Q.); paolo.manganotti@units.it (P.M.)

**Keywords:** amyotrophic lateral sclerosis, non-invasive brain stimulation, transcranial magnetic stimulation, transcranial electrical stimulation, transcranial ultrasonic stimulation, ALS, NIBS, TMS, TES, TUS

## Abstract

*Background and Objectives*: Amyotrophic lateral sclerosis (ALS) is a progressive neurodegenerative disease affecting the upper and lower motor neurons, with a bleak prognosis and few treatment options. Non-invasive brain stimulation (NIBS) techniques, such as repetitive transcranial magnetic stimulation (rTMS) and transcranial direct current stimulation (tDCS), represent emerging approaches aimed at modulating cortical hyperexcitability, a relevant pathogenetic mechanism in ALS. *Materials and Methods*: A systematic review of the literature was conducted following the PRISMA guidelines, exploring the Scopus and PubMed databases from April to June 2025 with terms related to ALS and NIBS. A total of 18 relevant studies were selected from the initial 708 articles, analysing stimulation protocols, clinical and neurophysiological outcomes, and associated biomarkers; their validity was assessed using the revised Cochrane risk-of-bias (RoB2) tool. *Results*: The selected studies were extremely heterogeneous, with NIBS techniques, including magnetic (rTMS, cTBS, tSMS) and electrical (tDCS) stimulation, showing variable effects. Low-frequency protocols (1 Hz rTMS) and cTBS showed a slight slowing of clinical progression, while prolonged home stimulation with tDCS and tSMS showed more significant improvements in terms of efficacy, tolerability, and adherence. The main limitations concern the heterogeneity of patients and protocols and the lack of standardised biomarkers, which is why the analysis remained at a descriptive level. The use of telemonitoring and caregiver training are essential to ensure safety and accessibility. *Conclusions*: NIBS represents a promising therapeutic approach for ALS, but further multicentre, standardised studies with prolonged follow-up are needed. Future strategies should include customisation of stimulation, combination with other therapies, and extension of application to pre-symptomatic phases.

## 1. Introduction

Amyotrophic lateral sclerosis (ALS) is a neurodegenerative disease affecting both upper and lower motor neurons (UMNs and LMNs), characterized by the progressive development of muscle weakness and motor dysfunction. In up to 50% of cases, extra-motor manifestations are also observed, including cognitive impairment, executive dysfunction, and language deficits; 10–15% of patients meet the diagnostic criteria for frontotemporal dementia (FTD) [1,2]. ALS has an estimated incidence of 1.75–3 per 100,000 persons per year and a prevalence of 10–12 per 100,000 in Europe, although significant geographical differences exist [2]. In approximately 90% of cases, ALS is sporadic, with no family history. The remaining 10% are classified as familial forms, typically exhibiting an autosomal dominant inheritance pattern. The most frequently implicated genes include *C9orf72* (the most common genetic cause of both ALS and FTD), *SOD1*, *TARDBP*, and *FUS*, among others [3,4,5]. Notably, *C9orf72* repeat expansions account for approximately 30–50% of familial ALS cases and 7–10% of sporadic cases, underlining their central role across ALS subtypes [2]. Symptoms usually develop during the fifth or sixth decade of life, but younger individuals can also be affected, particularly in familial cases. ALS carries a poor prognosis, with a median survival of approximately 3 years after symptom onset; death is most often due to respiratory failure or complications related to immobility [2,6,7].

**Figure 1:**
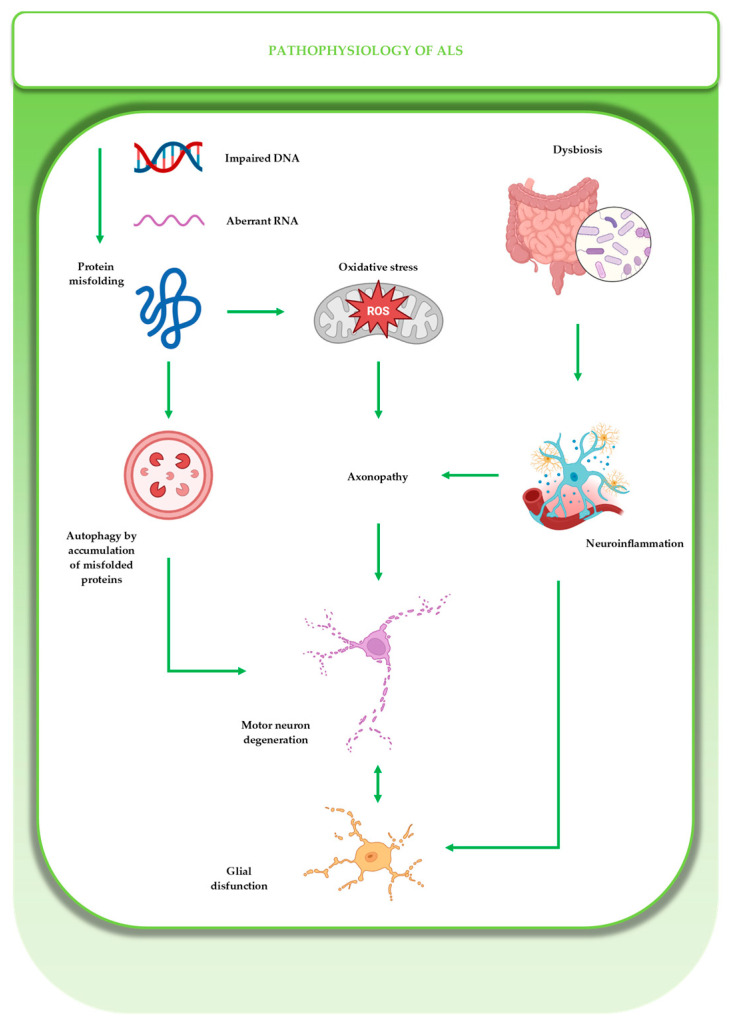
**Outline of ALS pathophysiology.** The precise mechanisms underlying motor neuron degeneration in amyotrophic lateral sclerosis (ALS) remain unclear but are thought to involve a multifactorial cascade of molecular and cellular dysfunctions. A key histopathological hallmark is the presence of intracellular aggregates of misfolded proteins, most commonly positive for TDP-43 and FUS, two nuclear RNA-binding proteins. Disruption of protein homeostasis correlates with cellular stress, oxidative damage, and ultimately leads to neuronal apoptosis. Other pathological mechanisms include impaired nucleocytoplasmic transport, defective DNA repair, and abnormal vesicle trafficking, all of which are associated with mitochondrial dysfunction and aberrant RNA processing. These disruptions further exacerbate oxidative stress, creating a vicious cycle that overwhelms cellular proteostasis. Certain genetic mutations have also been linked to cytoskeletal abnormalities and axonal transport deficits, contributing to microtubule destabilization and axonal degeneration. Motor neurons are particularly susceptible to excitotoxicity, a process supported by both neurophysiological evidence and the modest therapeutic efficacy of riluzole. Lastly, neuroinflammation, with activation of microglia and astrocytes, has been implicated in ALS pathogenesis. However, it remains uncertain whether glial activation represents a primary pathological driver or a secondary, potentially protective, response to neuronal injury.

The pathophysiological mechanisms underlying ALS are not yet fully understood. However, multiple cellular and molecular pathways have been implicated, including mitochondrial dysfunction, defects in axonal transport, abnormalities in RNA metabolism, accumulation of aberrant and misfolded proteins with impaired clearance, prion-like spreading mechanisms, excitotoxicity, oxidative stress, and neuroinflammation [8,9]. Among these, alterations in protein degradation, RNA metabolism, and axonal transport are considered the three principal pathological processes. This is supported by the fact that many of the genes associated with familial ALS encode proteins involved in these pathways (e.g., TDP-43 and FUS in RNA metabolism) [8,9] (Figure 1).

In recent decades, more than 40 randomized controlled trials failed to show a beneficial effect on disease progression or survival, and, currently, only symptomatic and a limited number of disease-modifying therapies are available [10]. Riluzole remains the only medication widely approved in Europe; it acts by reducing glutamatergic transmission and excitotoxicity through pre-synaptic inhibition of glutamate release or post-synaptic modulation of ionotropic glutamate receptors. It has been shown to provide a modest effect on median survival time, with an average increase of 2–3 months in randomized trials and up to 6 months in real-world studies, and current European guidelines advise its use in all ALS patients [11]. Edaravone is another medication that acts as a free radical scavenger preventing oxidative damage; it showed a slight slowing in disease progression at 6 months in a selected group of early affected patients without respiratory failure [12]. It has been approved for the treatment of ALS in the USA, Canada, Japan, South Korea, China and Switzerland, but it is not approved in the European Union, where the marketing application was withdrawn following a negative opinion from the EMA [13]. Among recently approved therapies, tofersen, an antisense oligonucleotide targeting *SOD1* mutations [5], received conditional approval in the USA in 2023 and in the European Union in May 2024, and was also approved in the UK in 2025. Other drugs are currently being studied, including verdiperstat, a selective myeloperoxidase inhibitor, although it failed to show efficacy in a recent platform trial [14], and several other compounds in early or confirmatory stages. For now, disease management still relies mainly on multidisciplinary care, consisting of symptomatic treatment options, including pharmacological and non-pharmacological interventions (stretching regimens and muscle relaxants for spasticity, nutritional counselling for dysphagia and weight loss, anxiolytic and antidepressant medications, sedative hypnotics, psychological support, etc.) [2,6,11].

In recent decades non-invasive brain stimulation (NIBS) has emerged as another possible therapeutic approach for neurodegenerative diseases, including ALS. Different techniques have been employed, particularly repetitive transcranial magnetic stimulation (rTMS) and transcranial direct current stimulation (tDCS), with different stimulation protocols. Also, invasive brain stimulation (IBS) of the brain cortex and the corticospinal tracts has been attempted. The main mechanism that justifies the application of these techniques is the phenomenon of cortical motoneuronal system’s hyperexcitability, which has been extensively described and studied. This phenomenon is strictly linked to the concept of cortical primacy in ALS pathogenesis, according to which the neurodegenerative process begins in the motor cortex and then spreads via corticofugal connections to α-motoneurons [15]. This hypothesis is supported by several observations: (1) muscle weakness being greater in districts with direct corticospinal projections, giving rise to ALS correlates like split-hand and split-leg syndromes, with districts not controlled by corticospinal tracts, like external oculomotor muscles and sphincter muscles, being spared; (2) the association between FTD and ALS; (3) TMS-derived neurophysiological evidence of hyperexcitable corticospinal circuits as reductions in SICI (short-interval intracortical inhibition), the resting motor threshold (RMT), and the cortical silent period (CSP) duration and an increase in motor evoked potential (MEP) amplitude; (4) evidence of cortical hyperexcitability preceding LMN dysfunction; (5) evidence of altered glutamatergic signals; and (6) presence of TDP-43 inclusions in specific cellular types (e.g., pyramidal cells in the primary motor cortex) [15]. This hypothesis is further supported by evidence that cortical hyperexcitability is useful to distinguish ALS patients from mimics and from healthy controls; thus, it has been proposed as a new diagnostic and pathogenetic biomarker to make an earlier diagnosis [16,17,18].

The first human study of NIBS was published in 1980 thanks to experiments by Merton and Morton who applied electrical stimuli on the human scalp using electrodes. They used a single high-voltage capacitive discharge that produced a current able to penetrate the brain and, by stimulating motor and visual cortex, produced muscle contraction on the contralateral side of the body and phosphenes, respectively. This method was named transcranial electrical stimulation (tES) and represented a new technique able to study CNS electrical properties but, unfortunately, it was not applicable for clinical and research purposes because of uncomfortable high-voltage current spreading on the scalp [19,20].

In 1985, Barker and colleagues introduced a new technique called transcranial magnetic stimulation (TMS), in which an electromagnetic coil placed on the scalp generates a magnetic field that induces an electric field capable of modulating the electrical activity of cortical neurons [21]. This phenomenon is based on Faraday’s law of electromagnetic induction, described in 1831 [19,20]. In this way, neurons can be influenced without the direct passage of electrical current through the skin, making the technique largely painless and highly suitable for both research and clinical applications.

The original use of TMS involved the application of single pulses over specific brain regions; however, over the following years, several stimulation paradigms and protocols were developed. These include repetitive TMS (rTMS), which delivers trains of repeated magnetic pulses; cortico-cortical paired associative stimulation (ccPAS), based on the timed delivery of paired stimuli to interconnected brain regions; and transcranial static magnetic stimulation (tSMS), which involves the application of a static magnetic field over a targeted brain area without inducing electrical currents [22,23].

At the end of the 20th century, another class of transcranial neuromodulation techniques gained attention. Building on early work with TMS, it was demonstrated that applying a low-intensity direct current to the human scalp could modulate the excitability threshold of cortical neurons, with effects dependent on the intensity and duration of the current [24,25]. When a direct current is used, the technique is referred to as transcranial direct current stimulation (tDCS), whereas the application of alternating current is termed transcranial alternating current stimulation (tACS). These techniques have since been expanded to include transcranial pulsed current stimulation (tPCS), a variation of tDCS in which current is delivered in a pulsed pattern, and transcranial random noise stimulation (tRNS), where current is applied at randomly varying frequencies. More recently, transcranial temporal interference stimulation (tTIS) has emerged, a technique that involves applying two high-frequency electrical fields outside the brain; their interference generates a low-frequency envelope capable of modulating deep brain structures while sparing the overlying cortical areas [26].

The third class of non-invasive brain stimulation (NIBS) techniques is known as transcranial ultrasonic stimulation (TUS), which is based on the application of ultrasound to the human scalp to influence neuronal activity. It includes low-intensity focused ultrasound stimulation (LiFUS) and transcranial pulse stimulation (TPS), the latter characterized by the delivery of pulsed ultrasound stimuli targeted to specific brain regions.

Unlike invasive brain stimulation techniques, NIBS methods offer the major advantage of being easily and transiently applicable, without requiring invasive electrode implantation surgery and its associated risks (e.g., the need for local or general anaesthesia, risk of infection at the implantation site, and the presence of a foreign body). This fundamental difference makes NIBS techniques not only more acceptable to participants, improving compliance, but also more scalable and feasible for both research and therapeutic applications.

Moreover, in the case of electrical stimulation, it is worth highlighting the possibility of administering these interventions in out-of-hospital settings. This allows patient, potentially affected by neurological disorders of varying severity, and their caregivers to take part in clinical trials or therapeutic programs from home, with minimal disruption to their daily activities [27,28].

Therefore, the aims of this systematic review are not only to summarise current knowledge on the effectiveness of NIBS techniques in ALS, identifying which methods are more appropriate than others (i.e., TMS vs. tES), but also to identify appropriate outcomes for evaluating effectiveness (i.e., MRC vs. dynamometer assessments) and to propose new ideas to guide future research in this field (i.e., combination of VR and NIBS).

## 2. Materials and Methods

The review was conducted and reported according to the Preferred Reporting Items for Systematic Reviews and Meta-Analyses (PRISMA) guidelines [29,30,31].

### 2.1. Selection Process and Eligibility Criteria

Study selection was performed independently by J.D.T., E.R., and M.Q., and any disagreements were resolved through discussion and consensus with two senior reviewers (P.M. and A.B.). Initially, titles and abstracts were screened, followed by full-text evaluation of studies that appeared potentially eligible. The literature search covered the period from 1998 to 2025, beginning with the publication of the first NIBS Ethics and Application Safety Guidelines [32,33,34].

Inclusion criteria were (i) studies involving adults (≥18 years) diagnosed with ALS of any stage; (ii) studies using any kind of NIBS (magnetic, electrical or ultrasonic stimulation); (iii) studies assessing quality of life, motor and cognitive outcomes; (iv) randomized controlled trials, prospective, or retrospective studies written in English. The exclusion criteria were (i) studies involving paediatric populations; (ii) studies using invasive brain stimulation; (iii) studies not reporting motor or cognitive outcomes or including non-ALS patients; (iv) non-peer-reviewed literature, including reviews, editorials, abstracts; (v) non-English publications.

### 2.2. Information Sources and Search Strategy

A comprehensive search of the relevant literature was performed using the Scopus and PubMed (Medline) electronic databases to identify potentially eligible articles. In addition, the reference lists of the included studies were manually screened to identify further relevant publications.

The systematic search was conducted between April and June 2025. The following search terms were used: [(ALS amyotrophic lateral sclerosis) OR (motor neuron disease) OR (ALS) OR amyotrophic lateral sclerosis] AND [(transcranial magnetic stimulation) OR (electrical stimulation of the brain) OR (magnetic field therapy) OR (TMS) OR (rTMS) OR (tSMS) OR (tDCS) OR (tACS) OR (tRNS) OR (tFUS) OR (LiFUS) OR (TPS) OR (TUS)].

**Figure 2:**
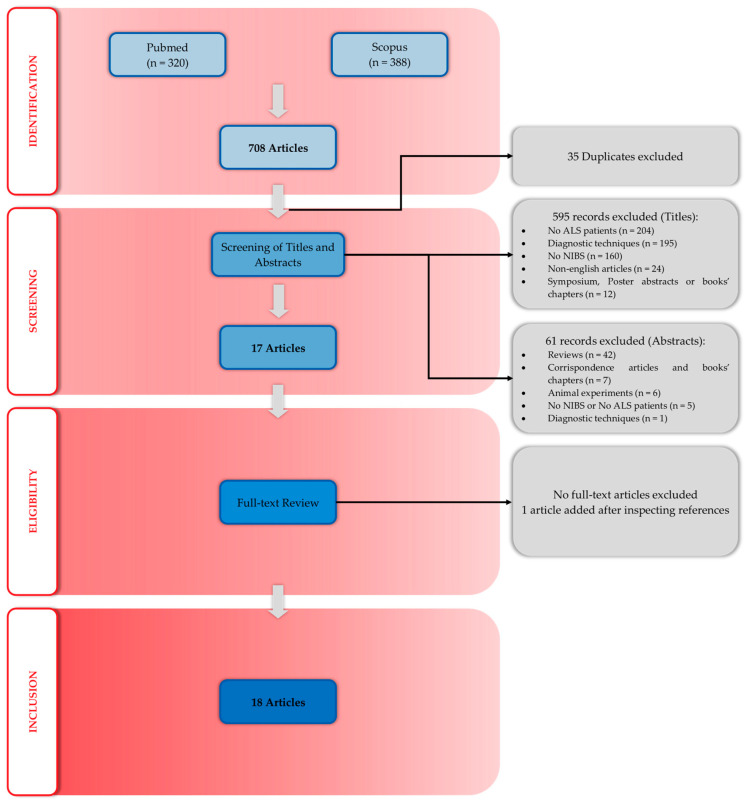
**PRISMA flow diagram of the study selection process**.

A total of 708 records were identified: 388 from Scopus and 320 from PubMed. After removing duplicates, 673 unique records remained. Of these, 17 articles passed the initial title and abstract screening and were further assessed for eligibility through full-text review. Furthermore, 1 additional article was identified by examining the reference lists of included studies, resulting in a total of 18 papers meeting the inclusion criteria and ultimately included in the review (Figure 2).

### 2.3. Data Collection Process and Items Selected

J.D.T extracted the data from included studies, while E.R. and M.Q. checked the extracted data. All variables included study design, participant demographics, intervention characteristics (including type, parameters, and timing of NIBS) were evaluated, and all the outcomes (i.e., scores on quality of life, motor performance and cognitive impairment) were considered for this review. Ultimately, the following data regarding the included studies was extracted: (i) design; (ii) type of NIBS technique; (iii) number of sessions; (iv) stimulation protocol details; (v) sample size. The following clinical variables were also extracted: (vi) quality of life (i.e., SF-36, ALSAQ-40, EQ5D5L, EQ-VAS); (vii) motor assessment; (i.e., MRC, ALSFRS-R, dynamometer, FSS, MVIC, Norris score, 10MWT, TUG, 6MWD); (viii) neurophysiological biomarkers (i.e., MEP, CMAP, RMT, SICI, ICF, EEG, CMCT); (ix) cognitive assessment (i.e., ECAS, BDI); (x) blood biomarkers (i.e., BDNF and NfL); (xi) caregiver burden (i.e., Zarit score, CBI).

All these data extracted were summarized and organized in tables. The three authors (J.D.T., E.R. and M.Q.) independently assessed study quality after full-text screening.

## 3. Pathophysiological Rationale for NIBS in ALS

**Figure 3:**
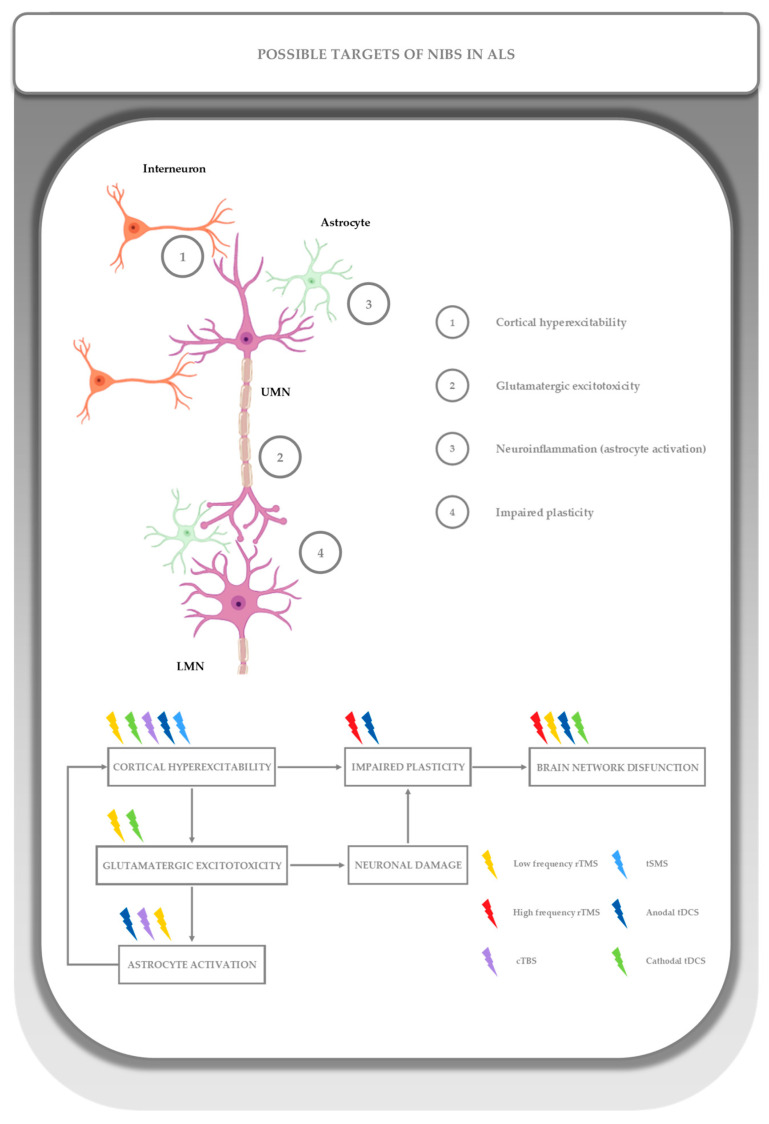
**Proposed mechanisms by which NIBS techniques target the pathophysiological features of ALS.** The figure summarizes the key mechanisms of neuronal damage in ALS and illustrates how these processes impair neuronal plasticity and disrupt communication between the motor network and other functionally connected brain regions. The lower portion of the image highlights the presumed or demonstrated sites of action of the main NIBS techniques used in ALS. UMN = upper motor neuron, LMN = lower motor neuron, rTMS = repetitive transcranial magnetic stimulation, cTBS = continuous theta burst stimulation, tSMS = transcranial static magnetic stimulation, tDCS = transcranial direct current stimulation.

To understand the rationale for applying non-invasive brain stimulation protocols in ALS, it is essential to consider the core neurophysiological and pathophysiological features of the disease. ALS is characterized by cortical hyperexcitability, microglial activation associated with inflammatory pathways, and alterations in neurotrophic signalling (Figure 3). These processes are not only central to disease progression but also represent potential therapeutic targets. NIBS techniques may influence each of these mechanisms, providing a rationale for their application and potentially serving as markers of treatment efficacy.

### 3.1. Cortical Excitability Impairment

ALS progresses through multiple stages; each associated with distinct alterations in cortical excitability. In the early phases, cortical hyperexcitability emerges as an epiphenomenon of motoneuron loss. However, it is now considered a driver of disease progression and neurodegeneration. If properly measured, cortical excitability may also serve as a prognostic marker. Over time, hyperexcitability transitions into hypoexcitability and ultimately inexcitability, mirroring the degeneration of the corticospinal tract, which is driven by progressive atrophy of the primary motor cortex and the loss of Betz cells in layer V of the cortex [35].

Cortical excitability can be assessed using TMS-based paradigms [36,37]. The state of hyperexcitability is primarily linked to an imbalance between GABAergic and glutamatergic neurotransmission, which are inversely related. These systems can be interrogated neurophysiologically using paired-pulse TMS techniques, particularly through short-interval intracortical inhibition (SICI) and intracortical facilitation (ICF) measurements [23,38,39,40]. A decrease in SICI, which reflects GABA_A_-mediated inhibitory circuits, combined with an increase in ICF, indicative of glutamatergic excitatory pathways, is considered a hallmark of cortical hyperexcitability in ALS [16,18,41,42,43,44,45,46,47,48,49,50,51,52,53,54,55,56]. Long-interval intracortical inhibition (LICI), mediated by GABA_B_ receptors, is also reduced in ALS patients and has been shown to correlate with greater disease severity [53]. In addition, a reduction in transcallosal inhibition has been observed, which is associated with more rapid disease progression and greater muscle weakness [57]. These findings further support the involvement of widespread inhibitory circuit dysfunction in ALS pathophysiology [23,58,59]. Recently, Ranieri et al. proposed that the MEP:CMAP ratio, derived through TMS, could serve as a prognostic biomarker in ALS. This ratio reflects the relationship between motor evoked potentials (MEP) and compound muscle action potentials (CMAP), which are indicative of UMN and LMN function, respectively. In cases where LMN involvement is predominant, the MEP:CMAP ratio tends to be higher, suggesting preserved cortical output despite peripheral motor unit loss. The reduction in SICI observed in these patients appears to be proportional to UMN involvement, implying that cortical hyperexcitability is an early and possibly initiating event. The MEP:CMAP ratio is also relatively easy to obtain from a technical standpoint and possesses the necessary characteristics to be a viable parameter for evaluating NIBS efficacy [35].

Both rTMS and tDCS have been investigated for their ability to modulate cortical excitability in ALS. Low-frequency rTMS and cTBS have been applied with the goal of reducing cortical hyperexcitability, theoretically restoring inhibitory control by normalising SICI and ICF. However, current evidence supporting these effects remains largely theoretical [37,38]. In contrast, tDCS has been shown to induce a significant increase in SICI without a corresponding change in ICF, suggesting a selective modulation of GABAergic neurotransmission and a potential to partially restore the excitatory–inhibitory balance [41,42,43].

### 3.2. Microglial Activation and Inflammatory Pathways

Among the key contributors to neurodegeneration in ALS are microglia and astrocytes. Although neuroinflammation is not considered the initiating factor in ALS, accumulating evidence suggests that disease progression is significantly amplified by the activation of both central and peripheral immune responses. Once activated, microglia release pro-inflammatory cytokines, such as interleukin-1β (IL-1β), interleukin-6 (IL-6), and tumour necrosis factor-alpha (TNF-α), as well as reactive oxygen species (ROS). Astrocytes also contribute to this pro-inflammatory environment through the production of ROS.

This inflammatory milieu promotes the recruitment and infiltration of the central nervous system by various immune cells, including monocytes, natural killer (NK) cells, and lymphocytes. The combined effects of cytokine release, ROS production, and immune cell infiltration contribute to progressive motor neuron damage. Notably, microglia appear to play a dual role: in the early stages of the disease, they exhibit a neuroprotective phenotype by producing anti-inflammatory cytokines such as IL-4 and IL-10 and releasing neurotrophic factors including insulin-like growth factor 1 (IGF-1). As the disease advances, however, microglia undergo a phenotypic shift toward a neurotoxic, pro-inflammatory state [60,61,62].

In this context, synaptic plasticity should be understood as the outcome of dynamic interactions not only between neurons but also involving glial cells and the cerebrovascular system. This neuro-glial-vascular unit plays a crucial role in maintaining homeostasis and responding to injury, and it is increasingly recognized as a target for therapeutic modulation in neurodegenerative diseases such as ALS [63]. Although human data remain limited, preclinical evidence suggests that NIBS techniques may modulate maladaptive glial responses. For instance, studies in ALS mouse models have demonstrated that rTMS can induce transcription of glial fibrillary acidic protein (GFAP), a marker of astrocyte activation that is dysregulated in ALS and other neurodegenerative diseases [64]. This modulation of GFAP expression suggests a potential role for rTMS in regulating astrocyte function and possibly attenuating neuroinflammation [63,65]. On the other hand, tDCS appears to influence glial physiology through a different mechanism by altering transmembrane potentials [66]. This change may restore the balance of neurotransmitter systems disrupted in ALS, contributing to neuroprotective effects. The modulation of glial excitability and ionic homeostasis by tDCS may also indirectly influence neuronal activity and synaptic plasticity, supporting its role in reshaping dysfunctional network dynamics in ALS [63,65].

### 3.3. Alteration of Neurotrophic Pathways

Neurotrophins (NTs) are a family of proteins that modulate intracellular tyrosine kinase pathways and play a key role in regulating synaptic plasticity. They support neuronal survival and differentiation, but under certain conditions, they can also promote apoptosis. NTs have been widely investigated in neurodegenerative diseases both as diagnostic and prognostic biomarkers and as potential therapeutic targets.

Within the context of non-invasive neuromodulation, neurotrophins such as brain-derived neurotrophic factor (BDNF), nerve growth factor (NGF), neurotrophin-3 (NT-3), and neurotrophin-4/5 (NT-4/5) have been studied as possible indicators of treatment efficacy. Among these, BDNF is by far the most extensively investigated [63]. In addition to classical neurotrophins, other neurotrophic factors that act predominantly on glial cells, such as glial cell line-derived neurotrophic factor (GDNF), neurturin (NRTN), persephin (PSPN), and artemin (ARTN), also contribute to neuronal survival and regeneration.

Several studies have evaluated serum BDNF levels in patients with ALS. Overall, BDNF appears to be a promising biomarker with prognostic and therapeutic relevance. Interestingly, BDNF concentrations in peripheral blood are often elevated in ALS patients, which is interpreted as a compensatory response to neurodegeneration [67,68].

Despite the interest in BDNF as a biomarker, current data on the effects of NIBS on serum levels of BDNF or other neurotrophins remain inconclusive. Preliminary findings suggest that BDNF levels may increase following repeated sessions of rTMS, with a possible correlation between stimulation duration and neurotrophin expression. However, evidence supporting similar effects for tES techniques, including tDCS or tACS, is lacking [69]. Furthermore, specific data on the modulation of neurotrophin levels in ALS patients undergoing NIBS are still insufficient. While some preclinical studies and indirect evidence suggest potential effects, well-designed clinical trials focusing on neurotrophic response in ALS are needed to establish any causal relationship or clinical significance [69,70,71,72].

### 3.4. Neuromodulatory Network for Neurological Diseases

One of the major challenges in diagnosing neurological disorders is the difficulty of mapping clinical symptoms to specifical anatomical regions of the brain. Over time, it has become evident that a single symptom may arise from lesions in multiple, spatially distinct areas [73]. This insight shifted the focus from isolated anatomical damage to dysfunction within broader neural networks thanks to the use of new neuroimaging techniques such as fMRI [74]. Lesion network mapping has increasingly been employed to identify brain networks associated with movement disorders such as hemichorea, parkinsonism, and cervical dystonia [75,76,77]. In neurodegenerative movement disorders, clinical progression likely reflects dynamic alterations across multiple functional circuits [78]. Molecular and metabolic imaging techniques, such as proton magnetic resonance spectroscopy (MRS) and ^18^F-FDG-PET, enable investigation beyond structural abnormalities, providing insights into neurotransmitter and metabolic dysfunction. The brainstem plays a central role in neuromodulatory circuits implicated in both motor and non-motor symptoms. Lesions affecting the dentato–rubro–olivary pathway, called the Guillain–Mollaret triangle, highlights how disruption of brainstem connectivity can result in different clinical syndromes [79]. In ALS, pathological involvement of the brainstem is well-established, representing the earliest stage of TDP-43 pathology according to Brettschneider’s staging system [80]. Despite this, neuroimaging studies have largely focused on corticospinal tract (CST) degeneration, with less attention given to other brainstem pathways. Recent diffusion tensor imaging (DTI) findings indicate focal atrophy in the medulla oblongata and degeneration in the CST and fronto-pontine tract, with reduced fractional anisotropy in these regions correlating with disease severity [81]. These results support a broader view of ALS as a multisystem disorder involving key brainstem circuits, with implications for both diagnosis and targeted NIBS strategies.

## 4. Overview of NIBS Modalities

In the realm of NIBS techniques, TMS and tES are the most frequently cited. However, the field of neuromodulation encompasses a much broader spectrum of approaches. Several attempts have been made to classify these techniques, but the most appropriate and widely accepted method is to categorize them based on the physical agent responsible for inducing neuromodulatory effects:

### 4.1. Magnetic Stimulation

**Figure 4:**
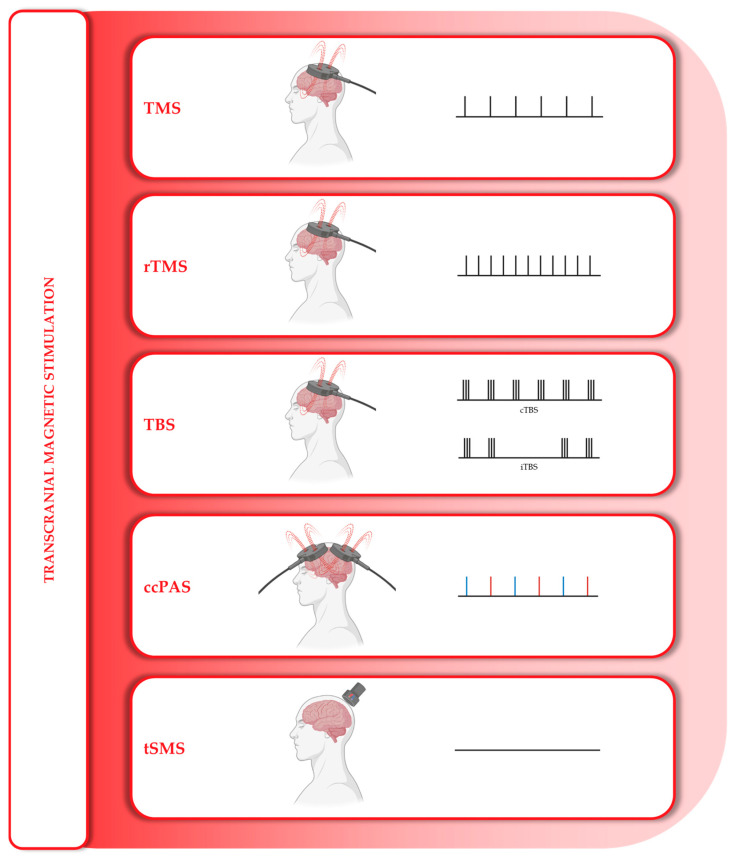
**Transcranial magnetic stimulation techniques.** On the left, an illustrative image depicts the different non-invasive brain stimulation (NIBS) techniques. On the right, a schematic graph shows the morphology of the electromagnetic wave induced by each technique, with stimulation frequency on the *x*-axis and amplitude on the *y*-axis. Techniques illustrated include TMS (transcranial magnetic stimulation), rTMS (repetitive transcranial magnetic stimulation), TBS (theta burst stimulation; c = continuous, i = intermittent), ccPAS (cortico-cortical paired associative stimulation), and tSMS (transcranial static magnetic stimulation).

This class is based on the use of magnetic fields, applied either in pulsed or static mode depending on the specific technique. TMS, rTMS, ccPAS, and tSMS all belong to this category (Figure 4).

Single-pulse TMS and its evolution, rTMS, involve the use of a coil placed on the scalp that delivers magnetic pulses capable of inducing an electric field sufficient to depolarize superficial axons and activate cortical neural networks. The efficacy of TMS depends on the current density generated in the brain, which in turn is influenced by several physical and biological parameters, including the type and orientation of the coil, the coil-to-cortex distance, the waveform of the magnetic pulse, stimulation intensity and frequency, as well as the alignment between the induced current lines and the orientation of excitable neural elements [82].

TMS delivers trains of brief, high-intensity magnetic pulses and can modulate cortical excitability depending on stimulation frequency: high-frequency stimulation (>5 Hz) tends to increase excitability, while low-frequency stimulation (<1 Hz) tends to reduce it. These effects are mediated by trans-synaptic modulation of neural circuits, primarily through neurotransmitter pathways [82,83,84]. The goal is to modulate maladaptive plasticity, such as reduced cortical plasticity or impaired connectivity, by restoring lost coherence and enhancing cortical reactivity and neurotransmitter release [83,85,86]. Due to its greater capacity to modulate neuronal activity, especially when administered over multiple consecutive days, rTMS has progressively replaced single-pulse TMS in most therapeutic protocols involving NIBS [84].

A specific form of rTMS is theta burst stimulation (TBS), which aims to replicate the endogenous theta rhythm, a natural neuronal firing pattern observed in the hippocampus. TBS typically consists of bursts of three stimuli at 50 Hz, repeated at 200 ms intervals (5 Hz). These bursts can be delivered either continuously (cTBS), producing effects akin to long-term depression (LTD), or intermittently (iTBS), inducing long-term potentiation (LTP)-like effects [84,87,88].

Another development in the field of magnetic stimulation is ccPAS, which involves the delivery of paired magnetic pulses to two functionally connected cortical areas at precisely defined inter-stimulus intervals. This protocol is designed to reshape network plasticity through Hebbian mechanisms, specifically spike-timing-dependent plasticity (STDP), which posits that the relative timing of pre- and postsynaptic activity determines whether a synapse is strengthened or weakened, thereby modulating the entire network [89,90,91].

In contrast to the techniques described above, tSMS employs a static magnetic field that does not generate an electric current. It involves the application of a cylindrical neodymium magnet over a targeted brain region, typically via a helmet-like support. The effect of tSMS appears to be inhibitory, possibly mediated by modulation of transmembrane ion channel activity [87,92,93,94]. Among magnetic stimulation techniques, tSMS is currently the only one with the potential for safe self-application in a home setting [87].

### 4.2. Electrical Stimulation

**Figure 5:**
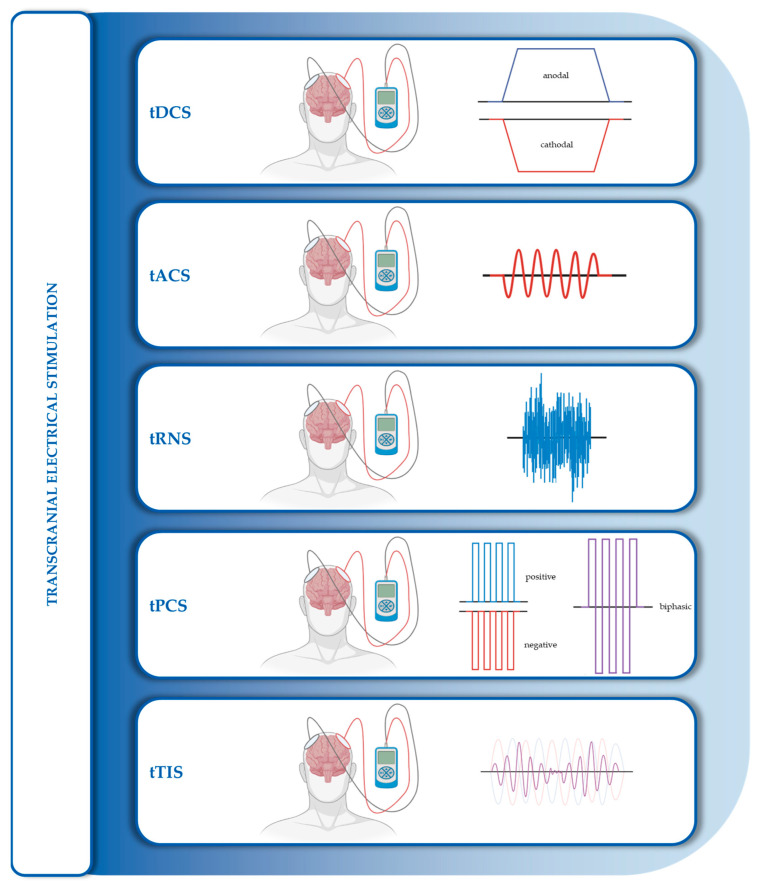
**Transcranial electrical stimulation techniques.** On the left, an illustrative image depicts the main NIBS techniques based on transcranial electrical stimulation. On the right, a schematic graph shows the morphology of the current waveform generated by each technique, with time on the *x*-axis and current amplitude on the *y*-axis. tDCS = transcranial direct current stimulation; tACS = transcranial alternating current stimulation; tRNS = transcranial random noise stimulation; tPCS = transcranial pulsed current stimulation; tTIS = transcranial temporal interference stimulation.

This class of NIBS techniques is based on the application of low-intensity direct or alternating electric currents delivered through electrodes placed on the scalp and connected to a stimulator. Techniques included in this group are tDCS, tACS, tRNS, and tTIS (Figure 5).

Transcranial direct current stimulation (tDCS) involves the application of a constant, low-intensity electrical current (typically 0.5–2 mA) to shift the resting membrane potential, inducing either depolarization or hyperpolarization depending on the direction of current flow relative to axonal orientation. The neuromodulatory effects are subthreshold and mediated through modulation of transmembrane ion conductance, membrane structure, cytoskeletal dynamics, and intracellular transport mechanisms, influencing both glutamatergic and GABAergic systems [95,96]. The effects of tDCS are often dichotomized: excitatory effects are typically associated with anodal stimulation, while inhibitory effects are linked to cathodal stimulation, at least in the motor cortex. However, this relationship is not universal, as several factors influence the outcome, including electric field characteristics (amplitude and intensity), electrode shape, neuronal orientation, extraneuronal tissue composition, and potential pathological or pharmacological influences [95,96]. These subthreshold changes in membrane polarization may ultimately lead to long-term potentiation (LTP) or long-term depression (LTD)-like effects.

Transcranial alternating current stimulation (tACS) applies a low-intensity alternating current (typically ≤ 2 mA) to modulate cortical excitability by entraining endogenous neural oscillations. The effects depend on the frequency of stimulation, which can modulate the amplitude, phase, and frequency of brain rhythms [97,98]. While most protocols do not exceed 2 mA, some studies have used intensities up to 3 mA [99,100,101,102]. The same biophysical and anatomical factors that modulate tDCS effects also influence tACS efficacy.

Transcranial pulsed current stimulation (tPCS) is a derivative of tDCS that delivers current in discrete pulses. It allows adjustment of pulse duration and inter-pulse interval and exists in two modes: monophasic and biphasic. In monophasic tPCS, pulse polarity is unidirectional, and the effects can be excitatory (positive polarity) or inhibitory (negative polarity), depending on the intended outcome. Biphasic tPCS, in contrast, delivers pulses of alternating polarity that cancel each other out, similarly to tACS. Stimulation frequencies typically range from 0.5 to 100 Hz, with current intensities between 0.7 and 2 mA. Its neuromodulatory mechanisms appear similar to those of tDCS in monophasic mode and to tACS in biphasic mode [103,104,105].

Transcranial random noise stimulation (tRNS) is a variant of tACS that applies an alternating current with randomly varying frequencies, typically within the range of 0.1–640 Hz, and with intensities of 1–2 mA and current densities below 1 A/m^2^. It is often subdivided into low-frequency tRNS (0.1–100 Hz) and high-frequency tRNS (100–640 Hz) [106,107]. The proposed mechanisms of action include increased excitability through facilitation of sodium channel opening and reduction of GABAergic inhibition, resulting in subthreshold neuronal depolarization. This state can lead to LTD-like plasticity through prolonged subthreshold excitation and decreased inhibition [106]. tRNS has also been associated with the concept of stochastic resonance, whereby the addition of white noise amplifies weak subthreshold signals, enhancing neural responsiveness. Only the components of the noise that match the frequency of the original signal are amplified, while unrelated noise components are filtered out [95,106,107,108]. Like tDCS, tACS, and tPCS, tRNS is well-suited for self-administered protocols in home-based settings [109].

Transcranial temporal interference stimulation (tTIS) is a novel approach and the only tES technique capable of targeting deep brain structures while sparing the overlying cortex. It is based on the principle of temporal interference, in which two high-frequency alternating electric fields (f > 1 kHz) are applied via scalp electrodes. When these fields differ slightly in frequency (Δf), their superimposition produces an amplitude-modulated envelope oscillating at the difference frequency. By carefully positioning the electrodes, this envelope can be directed to specific deep brain regions, where it modulates neural activity [26,110]. The focal modulation of tTIS depends on the spatial distribution and orientation of the two incident fields and may be further refined by combining multiple electrode pairs to enhance spatial selectivity [111,112,113].

### 4.3. Ultrasonic Stimulation

**Figure 6:**
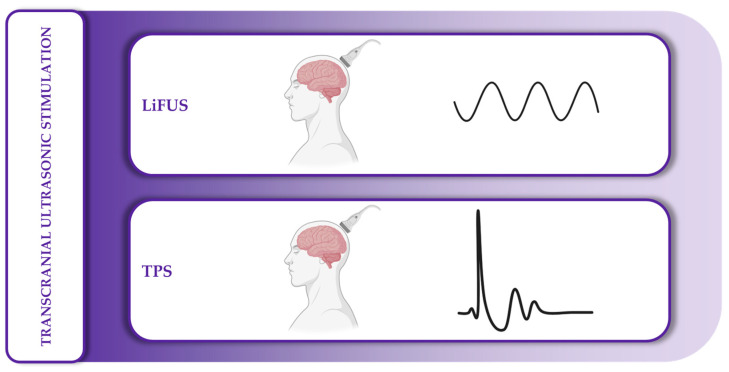
**Transcranial ultrasonic stimulation techniques.** On the left, an illustrative image depicts the main NIBS techniques based on ultrasound stimulation. On the right, a schematic graph shows the morphology of the acoustic wave generated by each technique, with time or frequency on the *x*-axis and acoustic pressure on the *y*-axis. LiFUS = low-intensity focused ultrasound stimulation, TPS = transcranial pulse stimulation.

This category of NIBS techniques is based on the use of ultrasonic waves applied to the scalp. LiFUS and TPS are the two main techniques belonging to this group (Figure 6).

Low-intensity focused ultrasound stimulation (LiFUS) is the non-invasive counterpart of high-intensity focused ultrasound stimulation (HiFUS), which is primarily used for ablative purposes in the central nervous system by generating temperature-controlled heat. While LiFUS and HiFUS are functionally distinct, a universally accepted cut-off between the two in terms of intensity or thermal output has yet to be clearly defined [114]. LiFUS exerts its neuromodulatory effects through transient mechanical and thermal perturbation of the neuronal membrane. This includes changes in membrane capacitance, altered permeability of ion channels that are gated mechanically, thermally, or chemically, and localized mechanical stress [59,70]. Ultrasound delivery is performed through a transducer that converts electrical energy into mechanical vibrations, producing ultrasonic waves. It is important to distinguish this from diagnostic ultrasound systems, which require both an emitting and a receiving component. In contrast, LiFUS uses only the transmitting element and is optimized for therapeutic rather than imaging purposes [114].

Transcranial pulse stimulation (TPS), unlike LiFUS, is based on the application of single, ultrashort (approximately 3 ms), repetitive mechano-acoustic pulses, typically delivered at frequencies ranging from 1 to 8 Hz. These shockwaves are thought to trigger a cascade of biochemical responses through a process known as mechanotransduction. TPS appears to modulate neural function by influencing neurotransmitter levels, including dopamine, serotonin, and GABA, and by promoting the release of various neuroactive and vasoactive molecules. These include vascular endothelial growth factor (VEGF), brain-derived neurotrophic factor (BDNF), glial cell line-derived neurotrophic factor (GDNF), and nitric oxide (NO). The combined effect of these molecular changes contributes to enhanced cerebral perfusion and the induction of neoangiogenesis, potentially supporting neuroplasticity and neuronal survival in targeted brain regions [84,115,116].

## 5. Clinical Evidence

NIBS techniques have been successfully applied for decades in both psychiatric and neurological settings, particularly in neurodegenerative diseases [34]. For about twenty years, they have been employed in the study of motor neuron diseases, especially ALS (see Table 1).

The first studies applying transcranial magnetic stimulation in ALS were conducted by Angelucci and Di Lazzaro and colleagues, who pioneered the use of rTMS at both low frequency (1 Hz) and high frequency (20 Hz) on the motor cortex of four patients, aiming to assess changes in muscle strength, disease progression and serum BDNF levels [71,117]. Angelucci et al. found that, after eight sessions, rTMS did not significantly alter BDNF levels in ALS patients, apart from a transient reduction following 20 Hz stimulation. Interestingly, a significant BDNF reduction was observed in healthy controls. This was interpreted as an epiphenomenon of cortical circuit degeneration in ALS, suggesting that the damaged circuitry may not be responsive to rTMS in the same way [71] Despite the small sample size, Di Lazzaro et al. reported faster disease progression in patients receiving high-frequency rTMS, as assessed by both MRC and Norris scores, compared to those receiving low-frequency stimulation [117]. The same group later applied cTBS in ALS, using a larger, placebo-controlled design. However, the results showed minimal effects on disease progression, and no significant changes were observed in secondary outcomes such as MRC, MVIC or BDNF. Extending the study from six to twelve months did not improve the findings, suggesting that baseline disease severity may play a crucial role in determining treatment response [72,118]. Zanette et al. employed a unique stimulation protocol at intermediate frequency (5 Hz), the only study using this approach, and were the first to evaluate quality of life and hand strength using SF-36 and dynamometry. They reported improvements in both metrics at the end of treatment, though the effects were not maintained at the two-week follow-up [119]. Di Lazzaro’s group later published a case study of a patient treated with cTBS for over two years, showing a marked slowdown in disease progression. This supported the hypothesis that treatment duration is a critical factor [120]. Munneke et al. were the first to correlate the clinical effects of rTMS with neurophysiological measures, specifically evaluating changes in cortical excitability via SICI and ICF. However, the only effects observed were a reduction in MEP amplitude and an increase in RMT, without significant changes in SICI or ICF. These effects were also not sustained over time [121]. More recently, Di Lazzaro’s group evaluated tSMS in ALS, assessing both clinical and neurophysiological endpoints. This study, which involved a relatively large sample, also enabled self-administered home stimulation, a strategy well suited to the progressive disability of ALS. Although no clinical improvements were detected, a reduction in MEP amplitude was observed during the first six months. Importantly, follow-up at 18 months revealed improved survival and prolonged tracheostomy-free time [92]. The most recent study using transcranial magnetic stimulation was conducted by Zheng et al., who applied 10 Hz rTMS over the DLPFC. This was the only study in the group targeting a cognitive outcome. They demonstrated cognitive improvement, as measured by the ECAS, with a robust sample size, marking the first such evidence in ALS [122].

Regarding transcranial electrical stimulation, the first study in ALS was by Quartarone and colleagues, who investigated the effects of tDCS on cortical excitability in both ALS patients and healthy controls. While cathodal tDCS reduced excitability and anodal tDCS increased it in healthy individuals, no effects were observed in ALS patients [123]. Similar findings were later reported by Munneke and Di Lazzaro, who found no significant effects of tDCS on either neurophysiological or clinical outcomes [124,125]. Madhavan et al. were the first to report motor improvement after tDCS, albeit in a single patient, showing enhanced performance in 10MWT and TUG. However, these results were not replicated by Sivaramakrishnan et al., who extended the protocol to two patients using a home-based device. Despite offering the possibility of self-administration, the results were negative [126,127]. A significant advancement in the field of transcranial electrical stimulation in ALS came from the work of Benussi and colleagues, who explored a novel corticospinal tDCS montage. In their first randomized, double-blind, sham-controlled trial, they applied simultaneous anodal stimulation over both primary motor cortices and a cathodal electrode placed over the cervical spinal cord. The study demonstrated that this configuration was associated with clinically meaningful improvements. Specifically, patients receiving active stimulation showed a significant increase in global muscle strength, better quality of life, and reduced caregiver burden compared to those receiving sham stimulation. Importantly, these clinical improvements were paralleled by neurophysiological changes, including a normalization of intracortical excitability, as assessed through SICI and ICF, suggesting a biological substrate for the observed effects [128]. In a subsequent study using the same stimulation parameters and montage, the authors evaluated the impact of a second course of tDCS, delivered following an initial two-week protocol. This extended intervention confirmed and reinforced the initial findings, with further clinical benefits and the addition of a reduction in serum neurofilament light levels, a promising biomarker of neurodegeneration. Moreover, patients who underwent two active courses of stimulation appeared to show a survival advantage, suggesting potential disease-modifying effects [41]. The most recent electrical stimulation study was conducted by Madhavan et al., who confirmed the safety and feasibility of a home-based device following proper training. Their results showed a slowdown in motor deterioration, further supporting the utility of remote tDCS [109].

**Table 1:** Non-invasive brain stimulation studies in ALS. Organized by type of stimulation, magnetic and electrical (in chronological order from the most recent). rTMS, repetitive transcranial magnetic stimulation; tSMS, transcranial static magnetic stimulation; cTBS, continuous theta burst stimulation; tDCS, transcranial direct current stimulation; d, day/days; m, month/months; w, week/weeks; DLPFC, dorsolateral prefrontal cortex; (b), bilateral; (an), anodal; (cat), cathodal; (m), motor cortex; (s), cervical spine; ALS, amyotrophic lateral sclerosis; (c), controlled sample; h, healthy subjects; ALSFRS-R, amyotrophic lateral sclerosis functional rating scale-revised; ECAS, Edinburgh cognitive and behavioural ALS screen; NfL, neurofilament light chain; MEP, motor evoked potentials; CMAP, compound muscle action potential; SICI, short-latency intracortical inhibition; ICF, intracortical facilitation; CMCT, central motor conduction time; MRC, medical research council scale for muscle strength; BDNF, brain-derived neurotrophic factor; FSS, functional system score; SF-36, 36-item short form health survey; MVIC, maximum voluntary isometric contraction; AP, average power; ROM, range of motion; ER, endurance ratio; ALSAQ-40, amyotrophic lateral sclerosis assessment questionnaire-40-item scale; CBI, caregiver burden inventory; EQ5DL5L, 5-level EuroQol-5D version; EQVAS, EuroQol-visual analogue scale; 10MWT, 10 min walk test; TUG, timed up-and-go test; 6MWD, 6 min walk distance; BDI, Beck’s depression inventory.

	Technique	Protocol	Stimulation Site	Dosage	Sample	Assessments	Main Findings
***Magnetic Stimulation***							
Zheng W. et al. (2025) [122]	rTMS	5 d/w, 4 w	DLPFC	10 Hz	80 ALS (c)	ALSFRS-R; ECAS; NfL; Zarit score	Significant short-term cognitive improvement and caregiver burden
Di Lazzaro V. et al. (2024) [92]	tSMS	3 times daily, 3 + 6 m	Motor cortex	0.12–0.20 T, 20 min × 2	40 ALS (c)	ALSFRS-R; MEP	Significantly improved tracheostomy-free survival (at 18 months)
Di Lazzaro V. et al. (2014) [129]	cTBS	5 d/m, 5/10/12 m	Motor cortex (b)	50 Hz	3 ALS	ALSFRS-R	Slowing disease progression
Munneke M. et al. (2013) [121]	cTBS	5 d	Motor cortex	50 Hz	10 ALS + 10 h	CMAP; RMT; SICI; ICF; EEG	Reduction in cortical excitability (MEP, RMT), which is not maintained at follow-up
Di Lazzaro V. et al. (2010) [120]	cTBS	5 d/m, 26 m	Motor cortex (b)	50 Hz	1 ALS	ALSFRS-R; MEP; CMCT	Slowing disease progression
Di Lazzaro V. et al. (2009) [72]	cTBS	5 d/m, 12 m	Motor cortex (b)	50 Hz	20 ALS (c)	ALSFRS-R; MRC; BDNF	No significant changes
Zanette G. et al. (2008) [119]	rTMS	5 d/w, 2 w	Motor cortex (b)	5 Hz	10 ALS (c)	ALSFRS-R; MRC; FSS; SF-36; MVIC; Dynamometer (AP, ROM, ER)	Increased grip strength and an improvement in SF-36, which are not maintained at follow-up (2 weeks)
Di Lazzaro V. et al. (2006) [118]	cTBS	5 d/m, 6 m	Motor cortex (b)	50 Hz	20 ALS (c)	ALSFRS-R; MRC; MVIC; BDNF	Slowing disease progression
Di Lazzaro V. et al. (2004) [117]	rTMS	10 d/2 w every 4 m; 25/30 m	Motor cortex (b)	1 Hz	2 ALS	MRC; Norris	Slowing disease progression
		8 d/m; 2/3 m		20 Hz	2 ALS		Faster disease progression
Angelucci F. et al. (2004) [71]	rTMS	8 d	Motor cortex	1 Hz, 20 Hz	4 ALS + 10 h	BDNF	No significant changes in serum BDNF levels in ALS subjects. Significant reduction healthy subjects
***Electrical Stimulation***							
Madhavan S. et al. (2025) [109]	tDCS	3 d/w, 12 w × 2	Motor cortex (an)	2 mA, 20 min	14 ALS (c)	ALSFRS-R	Slowing motor impairment
Benussi A. et al. (2023) [41]	tDCS	5 d/w, 2 w × 2	Motor cortex (an); cervical spine (cat)	2 mA (m), 4 mA (s); 20 min	31 ALS	ALSAQ-40; ALSFRS-R; CBI; EQ5D5L; EQ-VAS; ICF; MRC; NfL; SICI	Improvement in all parameters (confirmation of controlled phase results)
Sivaramakrishnan A. et al. (2019) [127]	tDCS	3 d/w, 8 w	Motor cortex (an)	2 mA, 20 min	2 ALS	ALSFRS-R; 10MWT; TUG; 6MWD; FSS; BDI	No significant changes
Benussi A. et al. (2019) [128]	tDCS	5 d/w, 2 w	Motor cortex (an); cervical spine (cat)	2 mA (m), 4 mA (s); 20 min	31 ALS (c)	ALSAQ-40; ALSFRS-R; CBI; EQ5D5L; EQ-VAS; ICF; MRC; NfL; SICI	Improvement in muscle strength, quality of life, survival and caregiver burden; but also, in neurophysiological parameters and serum reduction of NfL
Madhavan S. et al. (2018) [126]	tDCS	3 d/w, 4 w	Left Motor cortex (an + cat + sham)	2 mA, 20 min	1 ALS	ALSFRS-R; 10MWT; TUG; 6MWD; MEP	Slight motor improvement (10MWT and TUG)
Di Lazzaro V. et al. (2013) [125]	tDCS	1 d/m, 12 m	Motor cortex (cat) (b)	1 mA, 20 min	2 ALS	ALSFRS-R	No significant changes
Munneke M. et al. (2011) [124]	tDCS	1 d/w, 3 w	Left Motor cortex (cat)	1 mA, 7/11/15 min	10 ALS + 10 h	MEP; CMAP; SICI; ICF	No significant changes
Quartarone A. et al. (2007) [123]	tDCS	1 d	Motor cortex (an or cat)	1 mA, 7 min	8 ALS + 8 h	MEP; SICI; ICF	No significant changes

Therefore, by dividing the results according to outcomes, given the high heterogeneity of the data, the following observations can be made:

- Quality of life: Regarding rTMS, Zanette and colleagues, and regarding tDCS, Benussi and colleagues, demonstrated an improvement in perceived quality of life (QoL), although this aspect is subject to individual coping strategies and the psychosocial dynamics in which the patient is immersed. Furthermore, Benussi et al. also demonstrated an increase in overall survival. Di Lazzaro and colleagues demonstrated an improvement in quality of life (QoL), defined as the period without tracheostomy, in their study of tSMS application in ALS [41,92,119,128].
- Motor assessment: The most promising results were presented by Zanette and colleagues regarding rTMS, which showed a significant increase in grip strength, but it should also be noted that Di Lazzaro’s group demonstrated a tendency towards slowing the progression of the disease, assessed mainly with the ALSFRS-R, both after low-frequency rTMS and, above all, after cTBS. Regarding tDCS stimulation, Benussi and colleagues demonstrated an improvement in muscle strength, confirming the trend found by Madhavan’s group [41,117,118,119,120,126,128,129].
- Neurophysiological biomarkers: All studies that took them into consideration assessed cortical hyperexcitability. Among these studies, only Munneke’s cTBS study and Benussi’s tDCS study showed significant improvement; however, in the first one, this was not sustained in the long term [41,121,128].
- Cognitive assessment: This is an area that has rarely been investigated despite the motor component, so the results are very limited. The only satisfactory data comes from Zheng et al., who demonstrated short-term cognitive improvement following rTMS stimulation of the DLPFC (the only study to apply stimulation to this area). Sivaramakrishnan and his team considered evaluating the effects of stimulation on the BDI. Although the BDI predominantly represents a psychological domain, it showed no effect [122,127].
- Blood biomarkers: These have been considered since the first studies published in the literature, but while BDNF assessments have never yielded satisfactory results, tDCS stimulation performed by Benussi and colleagues appears to have induced a reduction in blood NfL levels, a result not observed by Zheng and colleagues with rTMS [41,71,72,118,122,128].
- Caregiver burden: This was assessed by Zheng and colleagues in their magnetic stimulation study, which showed an improvement in the Zarit score; similarly, Benussi and colleagues observed an improvement in the CBI assessment after electrical stimulation [41,122,128].

In summary, the application of NIBS in motor neuron disease, particularly ALS, is rapidly evolving. While current studies suggest promising potential for further development and clinical translation, the evidence remains heterogeneous in terms of patient cohorts, disease stages, stimulation protocols, and outcome measures. Therefore, these findings regarding efficacy should be interpreted with caution due to the heterogeneity and preliminary nature of the existing studies, as well as the limited duration of follow-up.

Despite this variability, existing data indicate that stimulation protocols suitable for home-based administration are both feasible and especially desirable given the progressive nature of ALS-related disability. It is also essential to continue investigating biomarkers such as plasma neurofilament light chain, whose clinical relevance is growing rapidly. Equally important is the need to integrate neurophysiological assessments to better understand treatment mechanisms and individual responses. To ensure robust evidence, future research should prioritise multicentre studies with sufficiently powered sample sizes. Finally, it should be noted that, to date, NIBS approaches based on ultrasound remain largely unexplored in the context of ALS and represent an important area for future investigation.

The validity of the included studies was evaluated by using the revised Cochrane risk-of-bias tool for randomized trials (RoB-2) as reported in the table below (see Table 2) [130]. RoB-2 is a tool that helps review authors assess the risk of bias arising from the inclusion of various studies in a review.

In conclusion, several research groups around the world are currently conducting, or planning to conduct, clinical studies aimed at evaluating the efficacy of various NIBS techniques in ALS. Abrahao and colleagues are investigating the effects of cTBS on cortical excitability by assessing neurophysiological parameters, magnetic resonance spectroscopy, as well as clinical and serum biomarkers, including neurofilament light chain [NCT05983211]. Amornvit and colleagues plan to explore the use of personalised rTMS in combination with mixed-reality exercise-based games (exergames) to slow disease progression and improve quality of life, assessing clinical, radiological, and neurophysiological outcomes [NCT07067229]. He and colleagues aim to evaluate the impact of rTMS on gait disturbances in ALS patients, with an integrated assessment of clinical, imaging, and serological parameters, including NfL levels [NCT06819358]. Fregonezi and colleagues are conducting a study on the effects of tDCS on pulmonary and neurological function, as well as neurophysiological measures and cortical tissue oxygenation assessed via near-infrared spectroscopy [NCT06719947]. Leon and colleagues are investigating the clinical efficacy of TPS, with a comprehensive evaluation of neurophysiological measures and serum biomarkers, including NfL [NCT06681610]. Finally, our group is conducting a clinical trial to evaluate the efficacy of home-based tDCS in slowing disease progression and improving quality of life, with parallel assessments of clinical and serum parameters, including NfL levels [NCT07006571].

## 6. Mechanistic Insights from Human Studies

### 6.1. Changes in TMS-Derived Excitability Measures

TMS enables non-invasive activation of the motor cortex and corticospinal pathways, both directly and trans-synaptically, providing a valuable tool for investigating physiological and pathological processes. Over recent years, it has increasingly emerged as a useful neurophysiological method to assess upper motor neuron (UMN) degeneration and its clinical correlates, offering both diagnostic and prognostic insights.

When a single TMS pulse is delivered over the motor cortex, the corticospinal system is activated, and a corresponding response can be recorded in a peripheral target muscle using surface electromyography. This response is termed the motor evoked potential (MEP). The minimal intensity required to elicit an MEP is referred to as the motor threshold (MT), which reflects the excitability of the motor system. MT is typically defined either as the lowest stimulation intensity needed to evoke an MEP of 50–100 μV in at least 50% of 10 consecutive trials at rest, or as the intensity required to consistently elicit MEPs of 0.2 mV amplitude [20,131]. Several studies have reported reduced MT values in patients with ALS, particularly during the early stages of the disease, and this finding has been interpreted as a marker of cortical hyperexcitability. Conversely, other investigations have described normal or increased MTs, or even an absence of motor responses due to cortical inexcitability. These inconsistencies likely reflect differences in disease stage at the time of assessment, underlying pathological heterogeneity, and variable progression rates. In particular, the absence of MEPs, reflecting a completely inexcitable motor cortex, has typically been associated with advanced stages of the disease, in which severe UMN degeneration is present [16,17,18]. Additionally, an increase in MEP amplitude has been reported in ALS patients but not in healthy controls or individuals with ALS-mimic syndromes [16,17,18].

Other single-pulse TMS-derived biomarkers include the cortical silent period (CSP) and cortical motor conduction time (CMCT), which provide additional insights into corticospinal tract integrity and cortical inhibitory processes. The CSP refers to a brief period of electromyographic silence following a suprathreshold TMS pulse applied to the primary motor cortex during voluntary muscle contraction. Its duration increases proportionally with stimulus intensity and is thought to reflect motor cortical inhibition, primarily mediated by GABA_B_ receptors. In ALS and other motor neuron diseases, CSP duration is frequently reduced or even absent, particularly in the early stages, consistent with a state of cortical hyperexcitability [43]. The CMCT measures the time required for a neural impulse to travel from the motor cortex to the spinal motor neurons via the corticospinal tract. It is calculated by subtracting the peripheral motor conduction time from the total MEP latency. Prolongation of CMCT has been observed in ALS and is believed to reflect demyelination or axonal damage of the corticospinal pathways. However, the correlation between CMCT prolongation and the degree of clinical neurodegeneration remains inconsistent, suggesting that CMCT may lack sensitivity as a longitudinal marker of disease progression [132].

A more sensitive method for detecting UMN dysfunction is the triple-stimulation technique (TST), a TMS-based protocol designed to overcome the variability and low reliability associated with standard MEP measurements, particularly amplitude assessments. The TST involves three sequential stimuli delivered at precisely defined interstimulus intervals. The first is a TMS pulse applied to the motor cortex, followed by a peripheral electrical stimulus to the distal segment of the nerve near the target muscle, and finally a second peripheral electrical stimulus applied proximally to the same nerve. When the motor neuron is successfully activated by TMS, the first cortical stimulus collides with the second antidromic peripheral impulse, cancelling each other, and the third stimulus then evokes a full CMAP. In contrast, if the TMS pulse fails to activate the motor neuron, due to UMN dysfunction, the second and third stimuli collide instead, leading to a reduced or absent CMAP. By comparing the resulting response with a normative reference, the proportion of functional corticospinal motor neurons can be estimated. TST has been shown to be more sensitive than MEP amplitude reduction or CMCT prolongation, and it can detect UMN involvement even in subclinical stages of ALS [133].

Unlike single-pulse TMS, paired-pulse TMS was developed to provide a more detailed assessment of the excitatory and inhibitory mechanisms of the cerebral cortex, particularly the role of cortical interneurons. This technique involves the delivery of two magnetic stimuli separated by a variable interstimulus interval (ISI). The first stimulus is typically subthreshold and does not elicit a motor evoked potential (MEP), while the second is suprathreshold and capable of producing a measurable MEP. The nature of the cortical response depends on the ISI.

When the ISI is short, typically 1–5 ms, the subthreshold conditioning stimulus leads to a suppression of the MEP induced by the test stimulus. This phenomenon, known as short-interval intracortical inhibition (SICI), is believed to reflect the activity of GABA_A_-mediated inhibitory interneurons and plays a physiological role in preventing the coactivation of unwanted muscles during voluntary movement [20]. Conversely, at longer ISIs ranging from 8 to 30 ms, the conditioning stimulus enhances the test stimulus response, resulting in increased MEP amplitude. This is termed intracortical facilitation (ICF) and is thought to involve excitatory glutamatergic interneurons within the primary motor cortex (M1), although spinal contributions have also been hypothesized [20,131].

In the context of ALS, numerous studies have demonstrated a marked reduction or complete loss of SICI, both in sporadic and familial forms of the disease, a feature that is not observed in ALS mimics [51,134]. This reduction in cortical inhibition is consistent with the concept of early cortical hyperexcitability in ALS pathophysiology. Similarly, when two suprathreshold stimuli are delivered at longer ISIs (typically 100–200 ms), a reduction in MEP amplitude occurs, a phenomenon known as long-interval intracortical inhibition (LICI). LICI is mediated by GABA_B_ receptors and has also been shown to be reduced in ALS patients, correlating with reduced SICI and greater disease severity [58,59]. Another paired-pulse measure of cortical excitability is short-interval intracortical facilitation (SICF), which involves two threshold or suprathreshold stimuli separated by ISIs of 1–5 ms. Unlike ICF, SICF is believed to reflect increased excitability of facilitatory interneuronal circuits. Elevated SICF has been reported in both classical and atypical ALS phenotypes, providing further evidence of altered excitatory–inhibitory balance in the motor cortex of ALS patients [135,136].

### 6.2. Neuroimaging Correlates

Recent advances in neuroimaging have enabled the identification of multiple radiological features associated with ALS. These include various magnetic resonance imaging (MRI) modalities, such as structural MRI, diffusion tensor imaging (DTI), and functional MRI, as well as positron emission tomography (PET). Each technique offers different insights into disease pathology and can reveal characteristic abnormalities, some of which may reflect underlying pathophysiological processes.

Structural MRI has historically revealed some of the earliest recognized alterations in ALS. Among the most common findings are hyperintensities along the corticospinal tracts (CSTs) on T2-weighted images, and ribbon-like bands of focal hypointensity in the precentral gyrus. While CST hyperintensities have been associated with advanced disease and proposed to reflect inflammatory mechanisms, their precise pathological significance remains unclear. Cortical atrophy, particularly affecting the motor cortex, has been consistently reported and is considered a hallmark of the disease. This atrophy reflects the well-established histopathological degeneration of upper motor neurons and has been proposed as a potential early marker of ALS. Additional structural abnormalities include atrophy of the thalamus, brainstem, and frontal and temporal lobes, findings more often associated with non-motor symptoms such as cognitive and behavioural impairment, particularly in ALS-FTD phenotypes. Although these radiological abnormalities have been validated in large patient cohorts, their limited sensitivity on an individual basis currently restricts their clinical utility [131].

Diffusion-weighted MRI (dMRI), particularly diffusion tensor imaging, offers a more sensitive assessment of white matter integrity by quantifying the diffusion of water molecules along fibre tracts. In ALS, reduced fractional anisotropy and increased mean diffusivity have been detected along the CSTs and in the corpus callosum. These findings are sensitive markers of disease, capable of distinguishing ALS from mimic syndromes and differentiating phenotypes based on the degree of upper motor neuron involvement [137,138]. Furthermore, reduced white matter integrity has been shown to correlate with clinical progression, making dMRI a promising candidate for disease monitoring [137].

Moreover, DTI parameters, particularly fractional anisotropy (FA), have been shown to closely correlate with clinical features, disease progression, and outcomes in ALS. Lower FA values were consistently associated with faster progression and poorer clinical prognosis [81]. These microstructural abnormalities have been identified in several key regions involved in both motor and extra-motor functions, including the motor cortex, precentral gyrus, central sulcus, insular cortex, cingulate gyrus and sulcus, and postcentral gyrus [81].

Proton magnetic resonance spectroscopy (MRS), applicable with both 3T and 7T MRI scanners, enables in vivo quantification of key neurochemical metabolites. In ALS patients, MRS has revealed decreased cortical levels of γ-aminobutyric acid (GABA), and increased glutamate concentrations compared to healthy controls [139,140]. A consistent finding across studies is the reduction of N-acetylaspartate (NAA), a marker of neuronal integrity, in regions such as the motor cortex and thalamus, reflecting early neuronal loss [81,141]. These reductions frequently co-occur with elevated choline levels, indicative of increased membrane turnover and glial activation [142]. The decline in NAA within the motor cortex and corticospinal tract (CST) has been shown to precede overt structural atrophy, challenging it as a sensitive early biomarker of disease activity.

These alterations are consistent with the excitotoxic and disinhibitory mechanisms implicated in ALS pathophysiology and may offer potential as non-invasive biomarkers of disease activity.

Functional imaging modalities, including functional MRI (fMRI) and positron emission tomography (PET), have provided valuable insights into the pathophysiological mechanisms underlying motor neuron diseases. fMRI, which detects blood-oxygen-level-dependent (BOLD) signal changes based on the coupling between neural activity and cerebral perfusion, has revealed significant alterations in the sensorimotor network of ALS patients. In early disease stages, increased connectivity within this network has been interpreted as a possible compensatory mechanism aimed at preserving function. These network changes have also been found to correlate with clinical manifestations, including motor impairment and cognitive decline [143]. Functional MRI (fMRI) studies provide an additional dimension by revealing disrupted connectivity not only within motor networks but also across extra-motor circuits, including regions involved in cognitive and behavioural functions, such as those comprising the resting-state networks [144]. ^18^F-FDG-PET studies have demonstrated characteristic patterns of hypometabolism in regions typically affected in ALS, such as the premotor and frontal cortices. Conversely, regions such as the brainstem and cervical spinal cord often display relative hypermetabolism, which may reflect gliosis or inflammatory processes [145]. Importantly, PET imaging offers the possibility of exploring metabolic and neuroinflammatory pathways more specifically through the use of targeted radioligands. Tracers that bind to proteins expressed by activated astrocytes and microglia have been used to localize focal inflammatory activity within the motor cortex and other disease-relevant areas. Furthermore, the use of GABA_A_ receptor ligands has enabled the investigation of inhibitory neurotransmission in ALS, potentially bridging neuroimaging findings with TMS-derived measures of cortical excitability [146,147].

### 6.3. Biomarkers

From a biological perspective, several studies have investigated molecular alterations associated with motor neuron degeneration, both to clarify underlying pathogenic mechanisms and to identify potential diagnostic and prognostic biomarkers. Among these, neurofilaments have emerged as the most promising biological markers, particularly in ALS.

Neurofilaments are structural proteins predominantly expressed in large, myelinated axons, composed of three subunits: light (NfL), medium (NfM), and heavy (NfH) chains. Upon axonal injury, these proteins are released into the extracellular space, where they can be detected in cerebrospinal fluid, serum, and plasma, serving as markers of axonal damage [148]. Accumulation of neurofilaments has been observed not only in ALS, but also in other neurodegenerative conditions such as Parkinson’s disease, Alzheimer’s disease, and progressive supranuclear palsy, suggesting their potential role as a general biomarker of neurodegeneration [148].

The light chain (NfL) and phosphorylated heavy chain (pNfH) are the most extensively studied isoforms in ALS. Elevated levels of NfL in CSF, serum, and plasma can distinguish ALS patients from healthy controls with high sensitivity and specificity. Although CSF concentrations are typically higher due to proximity to the central nervous system, NfL levels in the blood closely correlate with those in CSF, making blood-based assays a reliable and less invasive alternative [149]. Compared to other neurodegenerative diseases and ALS mimics, ALS patients exhibit higher levels of neurofilaments, which tend to remain stable throughout the disease course. Notably, higher NfL levels at symptom onset are associated with a more rapid disease progression and reduced survival, supporting their use as both diagnostic and prognostic markers [149,150,151,152].

Beyond diagnosis and prognosis, NfL levels are also being explored as surrogate biomarkers for treatment efficacy. In the VALOR study, a randomized controlled trial evaluating the antisense oligonucleotide Tofersen in patients with *SOD1*-mutated ALS, a significant reduction in plasma NfL levels was observed following treatment, although this was not paralleled by a clear clinical benefit [5]. Building on this finding and on accumulating evidence of a presymptomatic phase of ALS characterized by early NfL elevation prior to clinical onset [153], the ATLAS trial is now investigating Tofersen as a preventive intervention in asymptomatic *SOD1* mutation carriers, using NfL as a pharmacodynamic marker [154].

In addition to neurofilaments, neurotrophins such as brain-derived neurotrophic factor (BDNF) have also been studied as potential biomarkers in ALS. Some studies have reported elevated BDNF levels in ALS patients compared to healthy controls, which may reflect a compensatory response to ongoing neurodegeneration [63,67,68]. However, further research is needed to clarify the prognostic and therapeutic implications of neurotrophin modulation in ALS

### 6.4. Links Between Physiological Changes and Clinical Outcomes

The studies reviewed in this section consistently evaluated the effects of NIBS using a combination of clinical scales, functional scores such as the MRC sum score, and quality-of-life questionnaires. In addition to these subjective measures, several studies incorporated objective biomarkers, including electrophysiological parameters derived from TMS and, in some cases, biological markers such as neurofilament levels. Notably, however, none of the included studies assessed neuroimaging correlates as potential indicators of NIBS efficacy.

The lack of neuroimaging data may reflect the limited sensitivity of structural imaging techniques when applied at the individual level, or the relatively sparse integration of advanced MRI protocols into routine clinical settings, despite widespread availability. Furthermore, there remains uncertainty about whether NIBS can induce detectable macroscopic structural changes in the brain. In contrast, functional imaging, particularly PET, is a rapidly evolving field in ALS research and may provide valuable insights into the effects of NIBS on cortical excitability and metabolic activity, potentially offering a link between physiological and clinical responses. Furthermore, advanced MRI sequences such as MRS, DTI, and fMRI offer valuable insights into the relationship between structural and functional brain alterations and underlying neurophysiological circuits. These modalities hold significant potential for patient stratification and for identifying novel efficacy endpoints in clinical trials.

Regarding changes in TMS-derived excitability measures, several studies have reported important findings. In a recent study, Benussi and colleagues demonstrated that corticospinal tDCS can modulate intracortical circuits by restoring the balance between excitatory and inhibitory mechanisms. Specifically, after two weeks of active stimulation, an increase in short-interval intracortical inhibition (SICI) and a decrease in intracortical facilitation (ICF) were observed, suggesting normalization of cortical excitability [41].

In a sham-controlled pilot study, Madhavan and colleagues evaluated the effects of twelve tDCS sessions, applying anodal, cathodal, and sham stimulation, on motor cortex excitability using single-pulse TMS. Despite the patient being at an early stage of disease, no motor evoked potentials (MEPs) could be elicited either at baseline or after stimulation, reflecting a state of cortical inexcitability, in line with previous findings reporting motor cortex hypoexcitability in some ALS phenotypes [126].

Munneke and colleagues explored the effects of five consecutive days of cTBS in a small cohort of ALS patients. Their results showed a progressive reduction in MEP amplitude across sessions, with a significant increase in resting motor threshold (RMT). However, this modulation of corticospinal excitability was transient, as MEP amplitude returned to baseline one week after treatment. Importantly, there were no changes in SICI or ICF over the course of stimulation [121]. In a separate study, the same group assessed the impact of cathodal tDCS on corticospinal excitability in both healthy controls and ALS patients. While healthy participants showed a consistent reduction in MEP amplitude and changes in SICI after stimulation, ALS patients demonstrated minimal to no modulation in these parameters, suggesting impaired cortical responsiveness to inhibitory stimulation [124].

As described above, several studies have demonstrated that neurofilament light chain (NfL) levels increase with clinical disease onset in ALS, and more recently, this elevation has also been observed in asymptomatic carriers of pathogenic mutations. NfL concentrations at diagnosis have been shown to correlate with the rate of disease progression, with higher levels associated with more aggressive clinical trajectories, reinforcing the prognostic utility of this biomarker. In this context, Benussi and colleagues observed a significant reduction in serum NfL levels following corticospinal tDCS. This reduction became particularly evident over the full 48-week observation period, while no significant differences were observed at the 24-week mark. The authors interpreted this delayed but sustained effect as indicative of a potential neuroprotective role of this neuromodulatory intervention [41]. Similarly, Zheng and colleagues evaluated the effects of high-frequency rTMS in ALS patients over 6 and 12 months and found no statistically significant changes in plasma NfL levels compared to sham stimulation. Nonetheless, a trend towards deceleration in NfL elevation was noted in the active treatment group over the initial 6-month follow-up, suggesting a possible subclinical effect on axonal damage dynamics [122].

The potential impact of transcranial stimulation on brain-derived neurotrophic factor (BDNF) levels was first investigated by Angelucci and colleagues. In their study, 1 Hz rTMS applied to healthy subjects led to a progressive reduction in plasma BDNF levels, while no significant changes were observed in ALS patients stimulated with 1 Hz. Interestingly, 20 Hz rTMS induced a transient reduction in BDNF concentrations in ALS patients. These findings were interpreted as a possible modulation of BDNF production by rTMS through its effects on neuronal activity, although the results remained inconclusive [71]. Later, Di Lazzaro and colleagues examined the impact of a single five-day cycle of cTBS on plasma BDNF levels in a subgroup of ALS patients (five receiving active stimulation, five sham-treated). No significant changes were observed, and the authors highlighted the possibility that peripheral BDNF levels may not accurately reflect central nervous system concentrations [118]. In a subsequent study, the same group evaluated the effects of repetitive cTBS cycles, applied monthly over one year, on BDNF expression in peripheral blood mononuclear cells (PBMCs). Although a slight, non-significant increase in BDNF levels was detected on the third day of stimulation in the active group, no substantial changes were found after a single cycle [72].

Even if these are interesting results, it must be noted that they are based on different methodologies and reproducibility across them is lacking.

Studies evaluating the effects of NIBS techniques on NfL levels were conducted using four different techniques, each for every study: rTMS, cortico-spinal tDCS, cTBS and tDCS [121,122,124,128]. Sample size varied across studies, and the largest one was that of the study of Zheng et al. evaluating 80 patients, while in the other cases, the populations enrolled were smaller, with 31 in the study by Benussi et al., 10 in the one by Munneke et al., and 10 patients paired with 10 healthy controls in the second study by Munneke and colleagues [121,122,124,128]. The lack of multiple studies evaluating the same technique makes it impossible to compare results and generalize them; also, the small number of patients enrolled contributes to this. Furthermore, only one study compared the effect of NIBS between a group of healthy control subjects and a group of ALS patients, while the other three studies did not include this type of comparison, making the results obtained even weaker [124].

As far as BDNF is concerned, two different NIBS techniques have been investigated. Angelucci et al. evaluated the effects of rTMS, while Di Lazzaro and colleagues examined the impact of cTBS, first using a single five-day stimulation cycle and later employing repetitive cTBS cycles applied monthly over the course of one year [71,72,118]. The study by Angelucci et al. was unique in its design, enrolling 10 healthy controls and only 4 ALS patients. These characteristics make it challenging to generalize the findings or to compare them meaningfully with other NIBS studies. Similarly, although Di Lazzaro and colleagues assessed the same technique using two different protocols, to the best of our knowledge, no other studies have specifically evaluated the effects of cTBS on BDNF levels. In both cases, the small sample sizes, particularly the 20 patients included in Di Lazzaro’s studies, limit the robustness and generalizability of the results.

### 6.5. Links Between Genetic Background and NIBS Effectiveness

About 90% of ALS forms are sporadic, while the remaining 10% have an identified genetic cause. The four most common genetic subtypes of ALS are caused by mutations in *SOD1*, *FUS*, *C9orf72* and *TARDP* genes. For all these genes, different pathogenic mutations have been identified, but the mechanisms through which these mutations cause the disease are not yet fully understood. For example, most evidence has linked the pathogenicity of *SOD1* variants to a toxic gain of function, related to the propensity of mutated SOD1 protein to misfold, aggregate and spread in a prion-like fashion [155]. In *FUS*-mutated ALS, the primary driver of pathogenicity is thought to be the impaired nuclear localization of FUS protein, with a downstream of pathophysiological mechanisms and FUS protein’s toxic gain of function effect [156]. The most common genetic cause of ALS is the hexanucleotide repeat expansions in the *C9orf72* gene: in this case, pathogenesis derives from both a gain-of-function effect from the repeat DNA, sense and antisense repeat RNA, and dipeptide repeat proteins and a loss of function of the *C9orf72* protein effect, thus altering a diversity of cellular pathways [157]. *TARDBP*-ALS is another genetic form in which both gain- and loss-of-function effects are implicated: the protein TDP-43 mislocalizes from the nucleus to the cytoplasm leading to dysfunction of downstream pathways of RNA metabolism, proteostasis, mitochondrial function, oxidative stress, axonal transport, and local translation [158]. To make things more complex, the inheritance of these mutations is most often autosomal and typically with incomplete penetrance, and the influence of other factors such as environmental and epigenetic ones cannot be excluded.

As described above, cortical hyperexcitability is one of the main processes underlying ALS pathogenesis and it has been linked to synaptic alterations leading to overactivation of glutamate receptors, impaired inhibitory circuits, intrinsic motoneuron alterations, and astrocyte alterations; moreover, TDP-43 pathology may cause cell loss and synaptic alterations leading to compensatory hyperexcitability [87]. Even if the mechanisms underlying the development of cortical hyperexcitability remain to be fully elucidated, complex and dynamic changes within the cortical interneuronal networks have been demonstrated in animal models: for example, *SOD1*-G93A mice is an animal *SOD1*-mutated ALS’ model in which hyperexcitability was directed confirmed [159]. In ALS patients this phenomenon could be related to disinhibition of M1 due to degeneration of GABAergic circuits associated with increased cortical excitability secondary to excitatory cortical interneurons hyperactivity; this has been demonstrated by Vucic, Kiernan and colleagues who found a reduction in SICI in ALS patients [16,43,160].

As far as NIBS effects are concerned, different NIBS protocols interact in different ways with corticospinal circuitry. Specifically, protocols targeting indirect intracortical connections with CSNs, such as 1 Hz rTMS, PAS and cathodal tDCS, and protocols targeting direct connections with CSNs or CSNs themselves, such as cTBS, could be used, respectively, to compensate hyperexcitability related to hypoactive GABAergic interneurons or suppress intrinsic UMN excitability [87].

Given this link between hyperexcitability in *SOD1* mutation models and the effects of NIBS, it could be hypothesized that NIBS may be more effective in the forms of the disease where hyperexcitability and GABAergic dysfunction are prominent. The discovery of mutations most closely related to these neurophysiological alterations would make it possible to identify patients in whom a particular NIBS technique is most effective, leading to a more personalized and efficient approach. Furthermore, since pharmaceutical trials with antisense oligonucleotide capable to reduce the synthesis of mutated proteins (e.g., tofersen and jacifusen for *SOD1* and *FUS* mutated forms, respectively) are actually ongoing, it cannot be ruled out that in the future, with a more in-depth understanding of the neurophysiopathological mechanisms addressed by NIBS techniques, ALS therapy will become multimodal, comprising both a pharmacological approach and non-invasive neurostimulation. To make this possible, it is necessary to further investigate, on the one hand, the genetic heterogeneity of this disease and, on the other, all the possible mechanisms of NIBS that have not yet been fully explored [155,156].

## 7. Safety and Tolerability

This section addresses the safety and tolerability profile of non-invasive brain stimulation (NIBS) techniques, focusing specifically on magnetic and electrical stimulation methods, as these were the modalities included in the studies analysed.

NIBS techniques are globally considered safe and well tolerated in both patients and healthy individuals, with safety profiles supported by extensive literature and clinical experience. The only significant, albeit rare, adverse event is the occurrence of epileptic seizures, primarily associated with transcranial magnetic stimulation (TMS) and its variants [32,33,34,161].

In the case of magnetic stimulation, the risk of seizures is typically linked to high-frequency rTMS protocols or short inter-train intervals that may excessively modulate cortical excitability. Seizures can occur during stimulation trains or in the immediate post-stimulation period. Factors increasing the likelihood of seizure induction include a personal history of epilepsy, medications that lower the seizure threshold, or comorbid conditions such as stroke. The estimated crude seizure risk is approximately 1.4% in epilepsy patients and less than 1% in healthy controls. Generally, TMS is painless, whereas rTMS may cause discomfort at the site of stimulation. Pain intensity varies according to individual susceptibility, coil design, stimulation parameters, and target location. Other transient side effects include headache, neck pain, toothache, and scalp paraesthesia [32,161]. Importantly, reported cases of TMS-induced seizures are almost exclusively associated with protocols that failed to adhere to established international safety guidelines.

Electrical stimulation techniques, including tDCS, are also considered safe when used within recommended parameters (typically < 4 mA, ≤60 min per session). Common side effects are mild and transient, including sensations of itching or burning under the electrodes and occasional mild headaches. Rarely, superficial skin burns can occur, usually due to electrode dehydration or suboptimal contact with the skin. These can be prevented with appropriate skin preparation and electrode management [33].

### Safety and Tolerability in ALS Studies

Across the studies included in this review, no serious adverse events directly related to NIBS were reported in ALS patients. In magnetic stimulation studies, which collectively enrolled over 100 ALS participants, only a few minor adverse effects were noted. These included localized scalp discomfort in some patients undergoing rTMS and a single case of nausea during stimulation [122]. In the only study employing tSMS in ALS, among 32 participants, two reported headache and two experienced neck pain, the latter likely due to the physical weight of the stimulation device rather than the stimulation itself [92].

In studies involving tDCS, the most commonly reported side effects were consistent with those previously described in healthy populations: mild itching, tingling, and scalp sensations at the electrode sites. Notably, no serious adverse events were reported in any of these trials [109,124,127].

Overall, these data reaffirm the safety and tolerability of NIBS in ALS, supporting its continued investigation in both research and potential therapeutic settings.

## 8. Practical & Implementation Considerations

The application of neuromodulation techniques in ALS has progressively expanded, providing novel insights into their potential therapeutic roles. However, the clinical impact and feasibility of these techniques vary considerably depending on the specific NIBS modality used, the duration and intensity of stimulation protocols, and the outcomes assessed. Moreover, given the non-linear course of ALS and the often localized or subtle effects induced by NIBS interventions, exclusive reliance on the ALSFRS-R may lead to under-detection of clinically relevant changes and limit accurate stratification of patients suitable for NIBS approaches. To improve the sensitivity and overall interpretability of clinical trials in this heterogeneous population, it is crucial to integrate additional outcome and selection measures.

Table 1 provides a comprehensive summary of the studies conducted to date, detailing the stimulation parameters and primary endpoints explored across different protocols.

Low-frequency rTMS, particularly at 1 Hz, applied over the motor cortex, has been investigated with the aim of reducing the characteristic cortical hyperexcitability of ALS. Some studies have suggested a mild slowing in disease progression, whereas high-frequency rTMS protocols, such as 20 Hz, have instead been associated with symptom worsening [117]. A two-week protocol using 5 Hz rTMS, evaluated by Zanette and colleagues, yielded transient improvements in muscle strength and quality of life, effects that may be linked to BDNF-mediated mechanisms [119]. Studies using cTBS, intended to enhance cortical inhibition, have reported modest but statistically significant benefits in slowing clinical deterioration, although these findings are derived from small, underpowered trials with short follow-up periods and should therefore be interpreted cautiously [72,120,129,162,163].

More recently, a 10 Hz rTMS protocol targeting the dorsolateral prefrontal cortex (DLPFC) was tested in ALS patients with cognitive impairment. In this setting, the authors observed cognitive improvements at 12 months and a reduction in caregiver burden at 6 months post-treatment [122]. Finally, tSMS has emerged as a feasible home-based alternative, with Di Lazzaro et al. demonstrating the safety and potential efficacy of a thrice-daily, six-month protocol that increased tracheostomy-free survival [92].

In contrast, earlier studies investigating tDCS, particularly protocols using anodal or cathodal stimulation applied only over the motor cortex, produced limited clinical results [124,125,126,127]. However, a more recent and innovative approach combining anodal tDCS over the motor cortex with cathodal stimulation over the spinal cord has shown promising results. This dual-site montage was associated with improvements in muscle strength, reduction in the rate of functional decline and modulation of blood-based neurodegenerative biomarkers despite short-term follow-up [41].

These findings underscore the importance of prolonged and strategically targeted stimulation, particularly in protocols involving tSMS or dual site tDCS, which appear to offer the most practical, tolerable and potentially effective avenues for clinical benefit.

A key factor that emerges across these studies is the importance of cumulative stimulation dose, both in terms of protocol length and daily frequency. In this regard, home-based NIBS approaches provide a practical solution for delivering long-term treatment in a decentralized manner, thereby maximizing adherence and reducing the burden on both patients and caregivers. Hospital-based rTMS interventions, while supported by encouraging neurophysiological findings, are often constrained by limited accessibility and short treatment durations, which may limit their potential. Conversely, home-based protocols using tDCS and tSMS have demonstrated higher acceptability and compliance, supporting their long-term implementation in ALS patients. However, important challenges remain in terms of cost, management, caregiver training, and the regulatory disparities that influence access to these therapies, factors that still represent major barriers to widespread implementation across different regions.

Despite new evidence for the utility of NIBS in ALS, widespread clinical adoption is hindered by regulatory and logistical challenges. While the United States Food and Drug Administration (FDA) has approved rTMS for conditions such as major depressive disorder, migraine, and obsessive–compulsive disorder [164,165,166], no NIBS modality is currently approved for ALS, restricting their use to ethically approved clinical trials. In Europe, recent regulatory changes introduced under the Medical Device Regulation (MDR, Regulation 2017/745), specifically Annex XVI, have reclassified neuromodulation devices, including TMS and tES, as Class III or high-risk medical devices. This reclassification has imposed additional bureaucratic hurdles and ethical requirements that complicate the execution of research protocols and delay potential clinical translation. As highlighted by Antal and colleagues [167], these regulatory changes were implemented without thorough scientific review or adequate consultation with relevant experts and manufacturers, raising concerns about their validity and broader impact on innovation in neuromodulation.

An important factor for successful implementation is caregiver training. Home-based NIBS in ALS requires structured programs that include both theoretical instruction and hands-on practice in device use, correct electrode or coil placement, and adherence to stimulation protocols [92,109]. The integration of remote telemonitoring is also promising, but further validation is needed to ensure long-term safety, reliability, and usability in real-world settings.

Given the absence of curative treatments for ALS and the high cost of conventional care, such investments could provide meaningful improvements in patient access and quality of life.

## 9. Limitations of Current Evidence Base

While this review offers a comprehensive synthesis of the available evidence on non-invasive brain stimulation (NIBS) in amyotrophic lateral sclerosis (ALS), several limitations should be acknowledged. As a narrative review, the selection of studies was based on perceived relevance rather than predefined inclusion and exclusion criteria. This methodological approach introduces a risk of selection bias and may affect the generalizability of the conclusions drawn.

A prominent limitation across the reviewed studies is the marked heterogeneity in enrolled patient populations. Most studies included individuals at varying stages of disease progression and with heterogeneous clinical phenotypes. Factors such as small sample sizes, short follow-up durations, difficulties in recruitment, and inconsistent adherence to stimulation protocols further compound intra-group variability, thereby limiting statistical power and the ability to detect meaningful treatment effects.

Additionally, the variability in stimulation modalities, encompassing rTMS, tSMS, cTBS, and tDCS, along with differences in stimulation targets, intensities, durations, and anatomical montages, renders cross-study comparisons particularly challenging. These methodological inconsistencies hinder the development of standardized treatment paradigms and limit the ability to draw robust conclusions regarding efficacy.

The clinical heterogeneity observed among ALS patients also compromises the reliability of commonly used outcome measures in NIBS studies, particularly the ALSFRS-R. Although the ALSFRS-R remains the most widely adopted tool for assessing functional status in ALS, it lacks the sensitivity to detect subtle or short-term changes that may result from NIBS interventions. This limitation is especially pertinent considering the nonlinear and often biphasic progression pattern of ALS, characterized by rapid decline during early and terminal stages, with more gradual changes in between [168]. The frequent absence of significant ALSFRS-R changes in NIBS trials raises concerns regarding its adequacy as a standalone endpoint in this context.

A further limitation is the lack of harmonization in the use of objective neurophysiological and biological biomarkers across studies. Despite growing interest in the use of neurofilament light chain (NfL) as a prognostic biomarker, its application remains inconsistent [150,151,169,170]. Moreover, there is no standardized approach for stratifying ALS patients based on neurophysiological features, despite the emerging prognostic utility of parameters such as motor evoked potential (MEP), compound muscle action potential (CMAP), and particularly the MEP:CMAP ratio. The latter has recently been validated as a potential biomarker of cortical excitability and may serve as a useful stratification tool in future trials [35].

Another major limitation of the current literature is the insufficient integration of neuroimaging biomarkers into study designs. Most NIBS trials have focused primarily on clinical endpoints, such as ALSFRS-R or muscle strength, without systematically incorporating radiological markers, obtained from DTI, MRS, or fMRI sequences, that could provide objective insights into treatment-related neuroplastic changes. Similarly, patient selection has rarely been informed by imaging findings, despite evidence that structural and functional alterations in motor and extra-motor networks may influence responsiveness to NIBS.

In patients with advanced disease, often characterized by significant cognitive impairment and extensive cortical and spinal atrophy, neuromodulatory interventions may be less effective due to reduced neuroplasticity and diminished responsiveness to stimulation [35,171,172]. These anatomical and functional changes may also alter current distribution and reduce the efficacy of NIBS techniques. Consequently, the applicability and potential benefits of NIBS in this subgroup remain limited, and their inclusion in clinical trials requires careful consideration. Finally, a critical limitation affecting all these aspects is the lack of validated patient stratification strategies. ALS is a clinically and biologically heterogeneous disease, and without reliable prognostic or predictive biomarkers, it remains difficult to identify which patient subgroups may derive the greatest benefit from specific NIBS interventions. Addressing this issue is essential to designing efficient trials and ensuring that future clinical applications are both effective and equitable.

## 10. Future Directions

This review outlines the current state of non-invasive brain stimulation (NIBS) in ALS, highlighting the promising findings and the key limitations that currently prevent routine clinical adoption. Although several studies have reported potential benefits on clinical symptoms, neurophysiological parameters and biological markers, the substantial variability in study designs, stimulation protocols, patient cohorts and outcome measures limits the overall interpretability of results. Future research should focus on the standardization of stimulation protocols, extension of follow-up periods and the design of multicentre randomized controlled trials. In addition, integrating patient stratification based on clinical profiles, neurophysiological data and molecular biomarkers will be essential to improve treatment personalization and reproducibility [35,171,172].

In the early stages of ALS, diagnosis based solely on clinical criteria often leads to substantial delays, excluding many potentially eligible patients from timely therapeutic interventions. In this context, biomarkers, particularly neurophysiological markers such as the MEP:CMAP ratio, should be integrated into diagnostic workflows to enable earlier and more accurate identification of candidates suitable for NIBS interventions [35,41]. This ratio, easily obtained through routine TMS, represents a practical and reproducible marker of upper motor neuron involvement and could serve as a valuable stratification tool in future clinical trials.

Given the heterogeneity of ALS and the variability in patient responses to treatment, future research should also prioritize the development of adaptive stimulation protocols, new objective biomarkers and clinical scale for trial’s selection. Closed-loop systems capable of dynamically adjusting stimulation parameters in real time, based on feedback from neurophysiological biomarkers, offer a compelling avenue for achieving personalized and optimized neuromodulation strategies.

Moreover, combining NIBS with other therapeutic interventions may enhance its clinical efficacy. Future trials should investigate synergistic approaches that integrate neuromodulation with pharmacological agents, physical rehabilitation, or cognitive training programs. In particular, the combination of NIBS with virtual reality (VR) technologies has already shown potential in enhancing cognitive function and could be further explored in ALS patients, particularly those with cognitive or behavioural impairment [107,173]. There is also growing interest in applying NIBS in pre-symptomatic individuals carrying ALS-associated mutations with high penetrance, such as *SOD1*, *FUS*, *TARDBP*, and *C9orf72*. These individuals offer a unique opportunity to investigate the effects of early neuromodulatory interventions on delaying disease onset or modifying disease trajectory. Preliminary studies in carriers of frontotemporal dementia (FTD)-related mutations suggest that tDCS may influence early neurophysiological alterations, supporting the rationale for similar investigations in ALS [174,175].

Another emerging direction concerns the stimulation of spinal motor neurons in conjunction with cortical areas. Recent findings demonstrate that dual-site stimulation protocols, combining anodal tDCS over the motor cortex with cathodal tDCS over the spinal cord, can result in more robust clinical and biomarker improvements compared to cortical stimulation alone [41]. This approach may represent a critical step forward in targeting both upper and lower motor neuron dysfunction in ALS.

Finally, to facilitate the long-term application of NIBS in real-world settings, attention must be paid to safety, usability, and adherence, especially in the context of home-based protocols. From an economic perspective, while initial studies suggest that home-based stimulation methods such as tSMS and tDCS may reduce healthcare costs by limiting hospital visits and enabling decentralized treatment, the current evidence base remains limited and highly context dependent. Future studies should systematically evaluate cost-effectiveness across different healthcare systems. Targeted reimbursement strategies, particularly during training phases, are likely to be necessary for broader implementation but remain largely unaddressed in current policy frameworks. In this context, establishing targeted reimbursement policies, especially covering the initial training phase, could represent a practical and economically sustainable model.

## 11. Conclusions

NIBS methods have been investigated over the past two decades in ALS research, yielding valuable insights and opening promising avenues for future clinical application. As administration becomes increasingly feasible and accessible, NIBS represents a forward-looking area for large-scale, multicentre clinical trials. While the existing literature has demonstrated acceptable safety profiles and good tolerability across various NIBS modalities, the therapeutic efficacy remains uncertain due to methodological heterogeneity, small sample sizes, and short follow-up periods in most studies.

Among the modalities investigated, low-frequency rTMS has shown modest and transient effects in slowing clinical decline, though its practical application is limited by hospital-based delivery requirements. Moreover, tSMS, delivered through a home-based self-administration protocol, emerged as a promising approach. Recent advances in tDCS have also achieved the most comprehensive benefits with a combined protocol involving anodal stimulation over the motor cortex and simultaneous cathodal stimulation over the spinal cord. This approach has been associated with significant improvements in disease progression, albeit with short follow-up monitoring periods.

In this setting, new randomized, multicentre clinical trials are required with patient selection strategies that account for the clinical and biological heterogeneity of ALS. Integrating adjuvant therapies with virtual reality-based approaches may also enhance and extend the therapeutic benefits of neuromodulation protocols. Finally, future research should also focus on optimizing stimulation parameters, clinical patient selection, new radiological and neurophysiological biomarkers and improving home-based methods with standardized training for caregivers.

Through multidisciplinary collaboration, greater methodological rigor and the integration of complementary therapeutic strategies, the clinical translation of these neuro-modulation approaches may become feasible in future clinical practice. This would offer definitive insights into the true potential of NIBS techniques to modify disease progression, establishing a new therapeutic tool in the battle against ALS.

## Figures and Tables

**Table 2:** **Judgement of the risk of bias for each study included in the review.** Each domain listed at the top of the table corresponds to specific questions, which authors must answer with “Yes”, “Probably yes”, “Probably no”, “No” or “No information”. An algorithm assigns a specific risk of bias to each domain based on the answers to the individual questions. This results in risk assessments: (i) low risk of bias, (ii) some concerns, (iii) high risk of bias. The overall risk of bias is calculated considering that, to have an overall rating of “some concerns”, there must be only one domain rated as “some concerns” and no “high risk of bias”; otherwise, the study is rated as high risk overall. Obviously, if all responses are “low risk of bias”, the final rating is “low risk of bias”.

Study	Bias Arising from the Randomization Process	Bias Arising from Period and Carryover Effects (Crossover Trials Only)	Bias Due to Deviations from Intended Interventions	Bias Due to Missing Outcome Data	Bias in the Measurement of the Outcome	Bias in the Selection of the Reported Result	Overall Risk of Bias
Zheng W. et al. (2025) [122]	Low Risk	Not applicable	Some Concerns	Some Concerns	Low Risk	Low Risk	High Risk
Madhavan S. et al. (2025) [109]	High Risk	Not applicable	Low Risk	High Risk	Low Risk	Some Concerns	High Risk
Di Lazzaro V. et al. (2024) [92]	Low Risk	Not applicable	Some Concerns	Some Concerns	Low Risk	Some Concerns	High Risk
Benussi A. et al. (2023) [41]	Low Risk	Not applicable	Low Risk	Low Risk	Low Risk	Low Risk	Low Risk
Sivaramakrishnan A. et al. (2019) [127]	High Risk	Not applicable	High Risk	Low Risk	High Risk	High Risk	High Risk
Benussi A. et al. (2019) [128]	Low Risk	Not applicable	Low Risk	High Risk	Low Risk	Low Risk	High Risk
Madhavan S. et al. (2018) [126]	High Risk	Not applicable	High Risk	Low Risk	High Risk	High Risk	High Risk
Di Lazzaro V. et al. (2014) [129]	High Risk	Not applicable	High Risk	High Risk	High Risk	High Risk	High Risk
Munneke M. et al. (2013) [121]	High Risk	Not applicable	Some Concerns	Low Risk	Low Risk	Some Concerns	High Risk
Di Lazzaro V. et al. (2013) [125]	High Risk	Not applicable	High Risk	Low Risk	Some Concerns	High Risk	High Risk
Munneke M. et al. (2011) [124]	High Risk	Not applicable	Some Concerns	Low Risk	Low Risk	Some Concerns	High Risk
Di Lazzaro V. et al. (2010) [120]	High Risk	Not applicable	High Risk	Low Risk	Some Concerns	High Risk	High Risk
Di Lazzaro V. et al. (2009) [72]	Low Risk	Not applicable	Low Risk	Low Risk	Low Risk	Low Risk	Low Risk
Zanette G. et al. (2008) [119]	Low Risk	Not applicable	Low Risk	Low Risk	Low Risk	Low Risk	Low Risk
Quartarone A. et al. (2007) [123]	Some Concerns	Low Risk	Low Risk	Low Risk	Low Risk	Low Risk	Some Concerns
Di Lazzaro V. et al. (2006) [118]	Low Risk	Not applicable	Some Concerns	Some Concerns	Low Risk	Some Concerns	High Risk
Di Lazzaro V. et al. (2004) [117]	High Risk	Not applicable	High Risk	Low Risk	Some Concerns	Some Concerns	High Risk
Angelucci F. et al. (2004) [71]	High Risk	Some Concerns	Some Concerns	Low Risk	Low Risk	Some Concerns	High Risk

## Data Availability

No new data were created or analysed in this study. Data sharing is not applicable to this article.

## Abbreviations

The following abbreviations are used in this manuscript (arranged alphabetically):

6MWD	Six-minute walk distance
10MWT	Ten-minute walk test
^18^F-FDG	[^18^F] Fluorodeoxyglucose
AD	Alzheimer disease
ALS	Amyotrophic lateral sclerosis
ALSAQ-40	40-item ALS assessment questionnaire
ALSFRS-R	Amyotrophic lateral sclerosis functional rating scale—revised
AP	Average power
ARTN	Artemin
BDI	Beck’s depression inventory
BDNF	Brain-derived neurotrophic factor
*C9orf72*	Chromosome 9 open reading frame 72 (gene)
CBI	Caregiver burden inventory
ccPAS	Cortico-cortical paired associative stimulation
CMAP	Compound muscle action potential
CMCT	Central motor conduction time
CNS	Central nervous system
CSF	Cerebrospinal fluid
CSP	Cortical silent period
CST	Cortical-spinal tract
cTBS	Continuous theta burst stimulation
DLPFC	Dorsolateral prefrontal cortex
dMRI	Diffusion-weight magnetic resonance imaging
ECAS	Edinburgh cognitive and behavioural ALS screen
EEG	Electroencephalogram
EMG	Electromyogram
EQ-5D-5L	EuroQol group 5 dimensions 5 levels
EQ-VAS	EuroQol visual analogue scale
ER	Endurance ratio
FA	Fractional anisotropy
FDA	Food and drug administration
fMRI	Functional magnetic resonance imaging
FSS	Functional system score
FTD	Frontotemporal dementia
FUS	Fused in sarcoma
GABA	Gamma-aminobutyric acid
GDNF	Glial-derived neurotrophic factor
GenAI	Generative artificial intelligence
GFAP	Glial fibrillary acidic protein
HiFUS	High-intensity focused ultrasound stimulation
IBS	Invasive brain stimulation
ICF	Intracortical facilitation
IGF-1	Insulin-growth factor 1
ISI	Interstimulus intervals
iTBS	Intermittent theta burst stimulation
LICI	Long-interval intracortical inhibition
LiFUS	Low-intensity focused ultrasound stimulation
LTD	Long-term depression
LTP	Long-term potentiation
LMN	Lower motor neuron
M1	Primary motor cortex
MDR	Medical device regulation
MEP	Motor evoked potential
MND	Motor neuron disease
MRC	Medical research council scale for muscle strength
MRI	Magnetic resonance imaging
MRS	Proton magnetic resonance spectroscopy
MT	Motor threshold
MVIC	Maximum voluntary isometric contraction
NAA	N-acetylaspartate
NfH	Neurofilament heavy chain
NfL	Neurofilament light chain
NfM	Neurofilament medium chain
Nfs	Neurofilaments
NGF	Nerve growth factor
NIBS	Non-invasive brain stimulation
NIRS	Near-infrared spectroscopy
NO	Nitric oxide
NT	Neurotrophins
NRTN	Neurturin
PBMC	Peripheral blood mononuclear cells
PD	Parkinson disease
PET	Positron emission tomography
PMCT	Peripheral motor conduction time
pNfH	Phosphorylated heavy chain of neurofilaments
PRISMA	Preferred reporting items for systematic reviews and meta-analyses
PSP	Progressive supranuclear palsy
PSPN	Persephin
QoL	Quality of life
RCTs	Randomized multicentre trials
RMT	Resting motor threshold
RoB2	Revised Cochrane risk-of-bias tool
ROM	Range of motion
ROS	Reactive oxygen species
rTMS	Repetitive transcranial magnetic stimulation
SF-36	36-item short form health survey
SICI	Short-interval intracortical inhibition
SOD1	Superoxide dismutase 1
STDP	Spike-timing-dependent plasticity
tACS	Transcranial alternating current stimulation
TARDP	Transactive response DNA binding protein 43
TBS	Theta burst stimulation
tDCS	Transcranial direct current stimulation
TDP-43	Transactive response DNA binding protein 43 kDa
tES	Transcranial electrical stimulation
tFUS	Transcranial focused ultrasound stimulation
TMS	Transcranial magnetic stimulation
tPCS	Transcranial pulsed current stimulation
TPS	Transcranial pulse stimulation
tRNS	Transcranial random noise stimulation
tSMS	Transcranial static magnetic stimulation
TST	Triple-stimulation technique
tTIS	Transcranial temporal interference stimulation
TUG	Timed up-and-go test
TUS	Transcranial ultrasound stimulation
UMN	Upper motor neuron
VEGF	Vascular endothelial growth factor
VR	Virtual reality
